# Supplementary material for: Resolving thyroid lineage cell trajectories merging into a dual endocrine gland in mammals
Source: Nat Commun. 2026 May 25;17:6811. doi: 10.1038/s41467-026-73385-6 (PMC13385830; doi:10.1038/s41467-026-73385-6)
Supplement: Supplementary file 1 — Supplementary Information [file 41467_2026_73385_MOESM1_ESM.pdf]

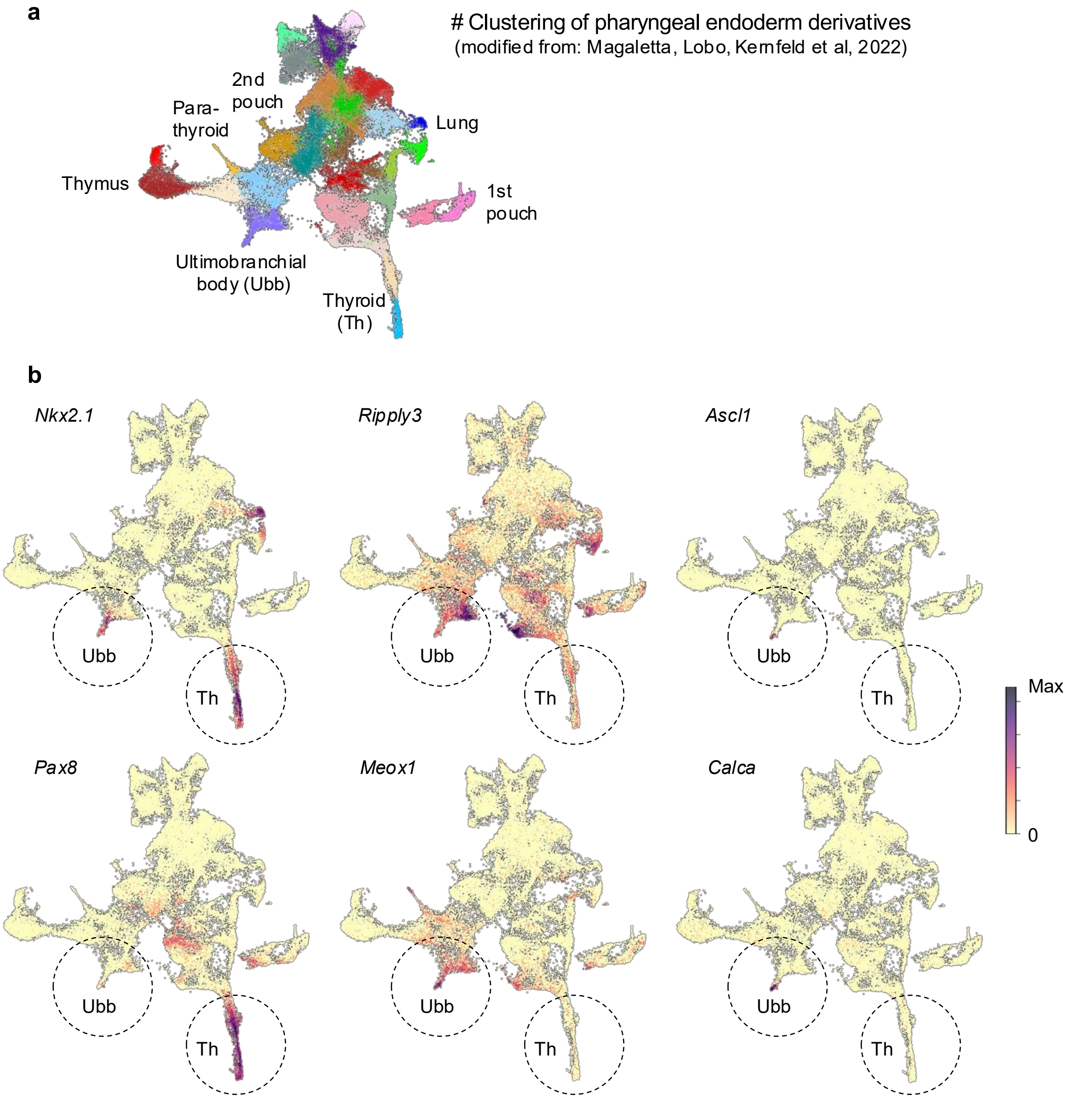

Supplementary Fig. 1. UMAP visualization of select thyroid and ultimobranchial body marker genes. Extracted from the single-cell transcriptome atlas previously generated from *Pax9*^{VENUS} embryos harvested daily between embryonic days (E)9.5 and E12.5 (Magaletta et al [14]). Supplementary to Fig. 1g, h.
a Overview depicting unsupervised clustering with the earlier less mature stages being distributed centrally in the UMAP embedding whereas the more mature and organ-specific cells are found in the peripheral clusters, as indicated. **b** Cluster-specific enrichment of the indicated genes of interest. Each dot represents a single cell in the gobal transcriptome space colored by average log2 normalized expression. Thyroid (Th) and ultimobranchial body (Ubb) clusters are encircled. Scalebar indicates log2-normalized expression.

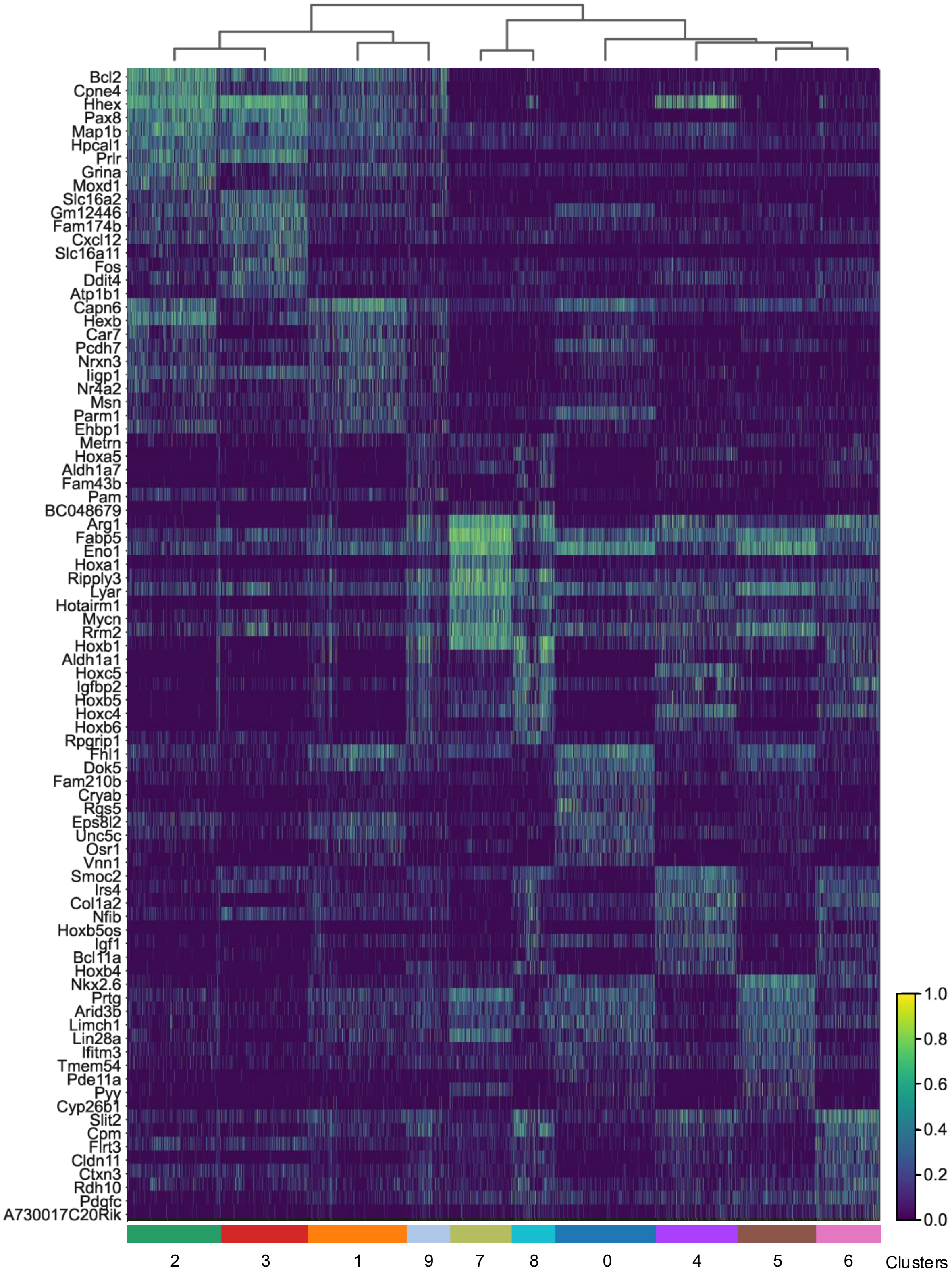

Supplementary Fig. 2. Clusters with thyroid and ultimobranchial lineage identities distinguished by enriched genes. Heatmap showing the scaled expression of the of top 10 upregulated genes in each of clusters 0-9 exclusively featuring an adjusted p-value <0.01, a log2-fold change >1.0, and being expressed in >20% of the cells in the cluster. Supplementary to Fig. 1i, k. Genes upregulated in multiple clusters are displayed corresponding to the cluster having maximum average log2-normalized expression of the gene. The dendrogram obtained from unsupervised hierarchical clustering is displayed. Scalebar indicates log2-normalized expression. Source data are provided in Supplementary Data 1.

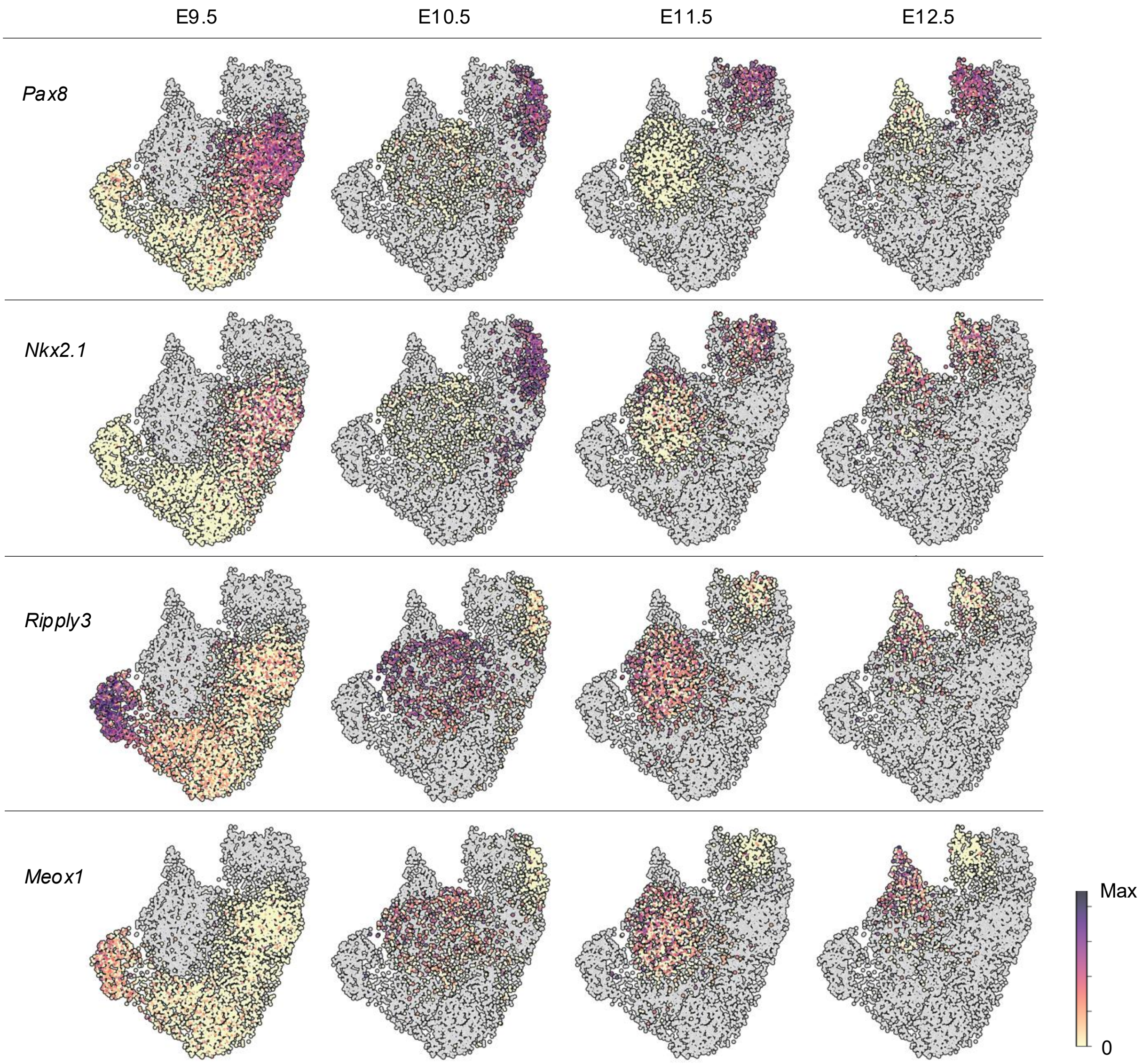

Supplementary Fig. 3. Single-cell mRNA expression of thyroid and ultimobranchial lineage marker genes across Eday. UMAP embedding showing expression patterns of *Pax8*, *Nkx2-1*, *Ripply3* and *Meox1* separately at each embryonic day between E9.5 and E12.5 (top panels). Supplementary to Fig. 1i-l. Cells are outlined and colored by the log2-normalized expression if they are present at the corresponding embryonic day. Gray outlined dots represent cells present in the dataset from timepoints other than the one indicated in the plot. Leiden cluster assignments with predicted thyroid lineage clusters 1-3 and Ubb clusters 4, 6 and 8, according to unsupervised global scRNAseq analysis, are shown for comparison (right panel). Scalebar indicates log2-normalized expression. Ubb ultimobranchial body.

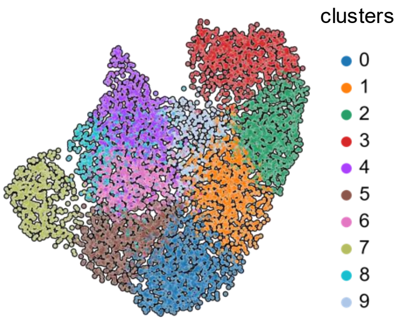

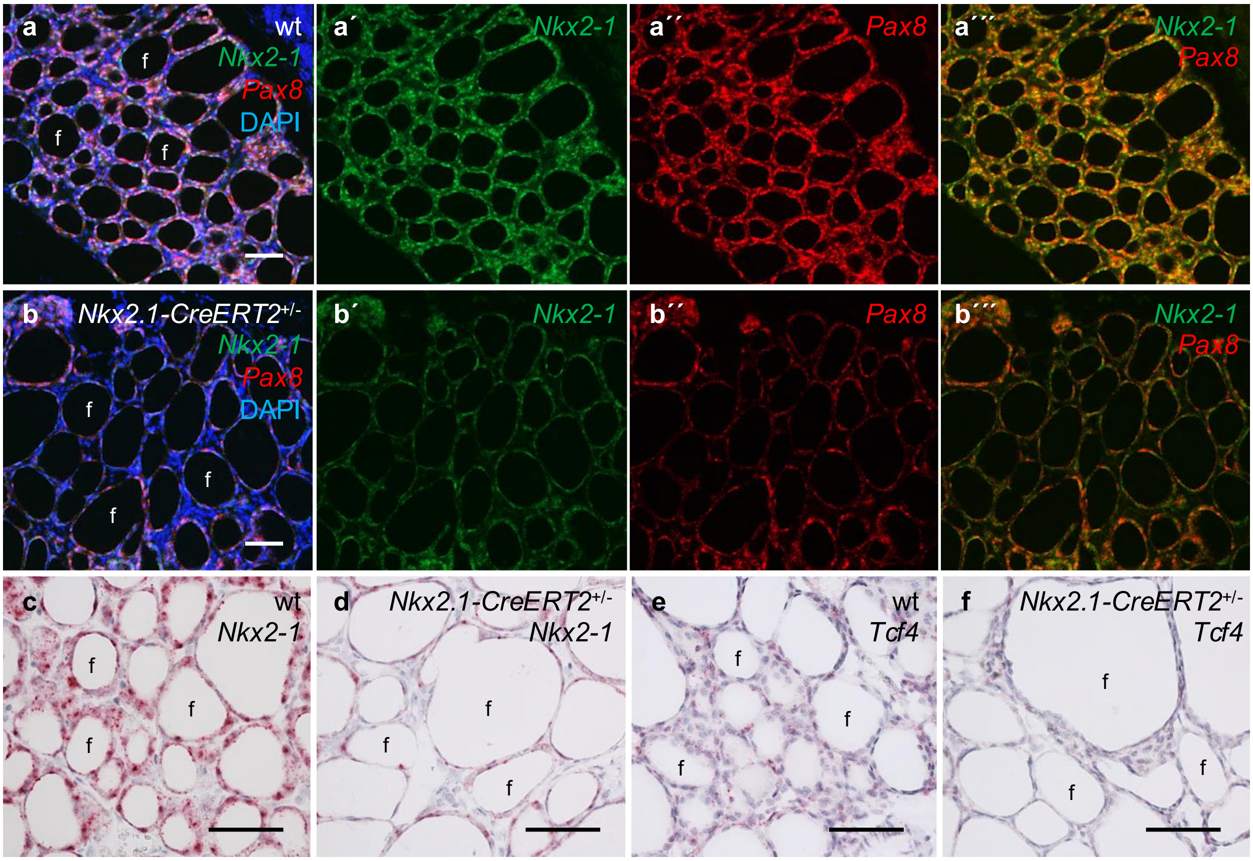

Supplementary Fig. 4. Downregulation of Pax8 and Tcf4 in heterozygous *Nkx2-1* deficient mouse thyroid cells *in vivo*. Thyroid FFPE sections of wildtype (wt) and heterozygous *Nkx2.1-CreERT2* mice (age: 6 months) were subjected to RNAscope analysis to validate the *Pax8* gene as a predicted target of Nkx2-1 according to Ordinary Fig. 3e and Supplementary Table 1. This mutant is equivalent to heterozygous Nkx2-1 null mice as evidenced by similarly altered follicular morphology. Multiplex fluorescence was applied for colocalization in (a, b) whereas histochemical detection allowed comparison of high and low abundance mRNA levels in (c-f). **a** Co-expression of *Nkx2-1* and *Pax8* in the majority of thyroid follicular cells. **b** Diminished expression of *Pax8* in cells with reduced *Nkx2-1* expression due induced inactivation of the Cre driver allele. **a´-a´´´, b´-b´´´** show single/double channel without DAPI nuclear staining for improved visualization. Note heterogenous expression of Nkx2-1 and Pax8 is evident among follicular cells in both normal and mutant thyroids. **c-f** Diminished expression of *Tcf4*, another predicted Nkx2-1 target gene (see Supplementary Table 1), in *Nkx2-1* haploinsufficient thyroid cells. f follicles. Scale bars: 100 μm.

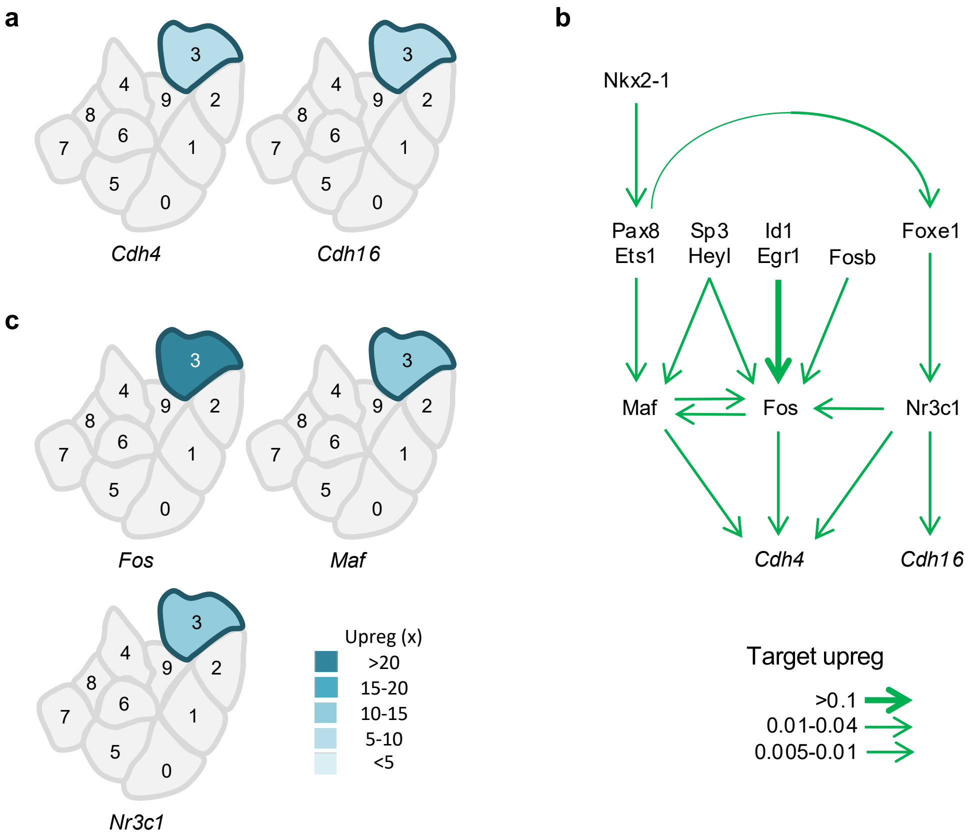

Supplementary Fig. 5. Enriched expression of cadherin-4 (R-cadherin) and cadherin-16 (Ksp-cadherin) and their predicted transcriptional regulation in embryonic thyroid cells undergoing differentiation. a Upregulation of *Cdh4* and *Cdh16* in cluster 3 only. Curated from lists comprising all enriched genes per cluster. **b** Predicted subnetwork of *Cdh4* and *Cdh16* regulation in thyroid lineage cells identified by CellOracle. Arrows indicate upregulation with arrow thickness representing mean cluster-specific GRN TF–target gene interaction scores. No TFs were found to downregulate these cadherins in the filtered thyroid and Ubb GRNs. **c** Cluster-specific upregulation of key TFs predicted to regulate *Cdh4* and *Cdh16* expression in cluster 3 cells. Supplementary to Fig. 3a-e. GRF gene regulatory network. Source data are provided in Supplementary Data 1, 5.

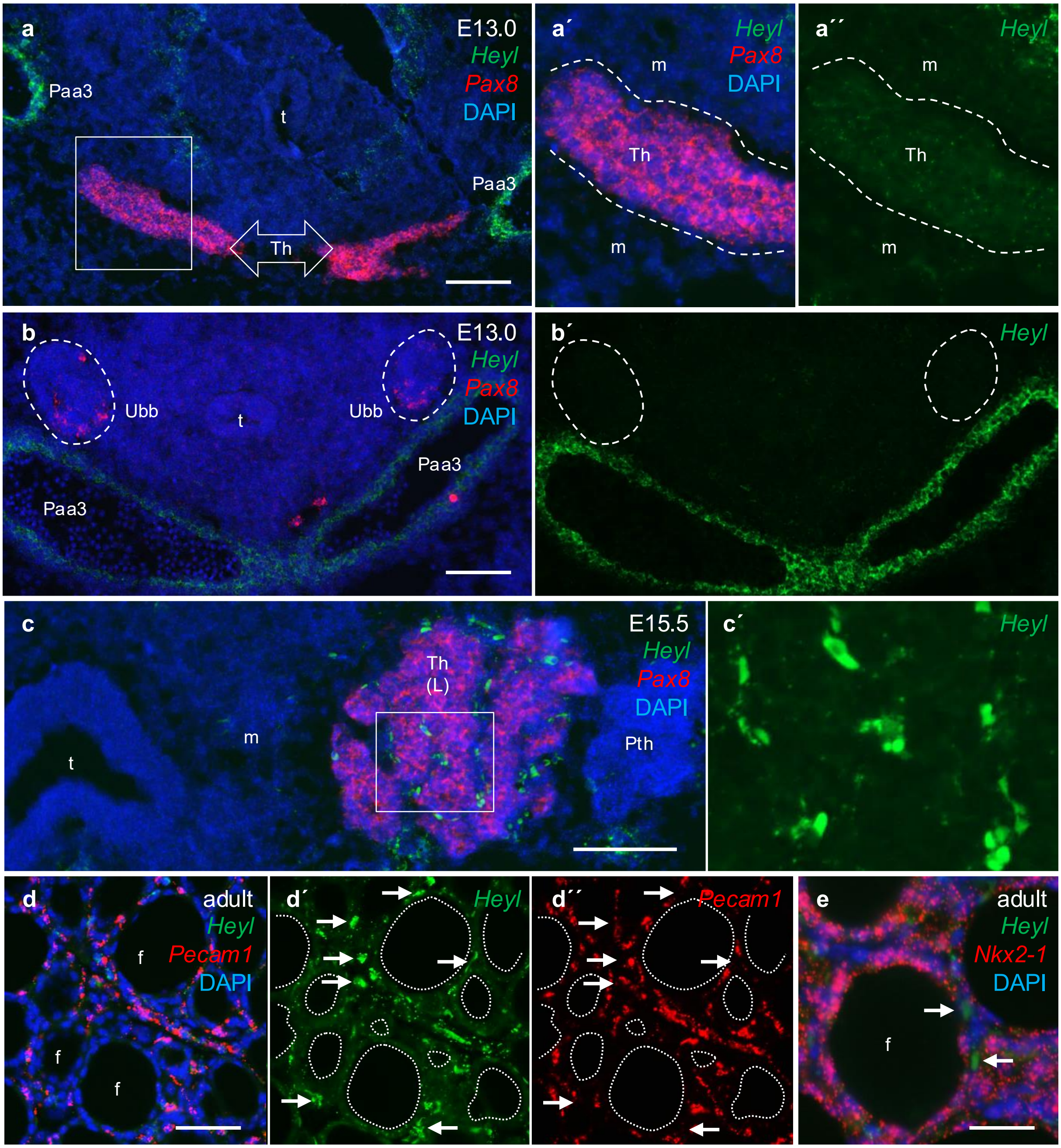

Supplementary Fig. 6. Heyl expression pattern in the embryonic and adult mouse thyroid gland. RNAscope Multiplex fluorescent analysis of *Heyl* vs *Pax8* (a-c), *Pecam1* (d) and *Nkx2-1* (e) expression was done to validate scRNAseq data presented in Ordinary Fig. 3. Single channels are shown in (a´´, b´, c´, d´, d´´) for improved clarity. **a** Bilateral growth of the midline thyroid primordium at E13.0; a´, a´´ show high power of boxed area in (a) with weak *Heyl*+ thyroid tissue encircled. **b** Ultimo-branchial bodies prior to fusion with the thyroid; b´ shows the Ubbs are uniformly *Heyl* negative. Note scattered *Pax8*+ Ubb cells confirming immunofluorescent data in Ordinary Fig. 2k. **c** Accumulation of *Heyl*+ interstitial cells in the embryonic thyroid lobe; c´ shows high power of boxed area in (c). **d** Differential *Heyl* expression in the adult thyroid gland: weak in follicular cells; moderate in microvessels; strong in scattered, *Pecam1* negative interstitial cells indicated by arrows in (d´-d´´) in which follicle lumina are outlined for clarity. **e** *Heyl*+ interstitial cells are Nkx2-1 negative (arrows). Th thyroid, Pth parathyroid, Ubb ultimobranchial body, Paa Pharyngeal arch artery, t trachea, m mesenchyme, f follicle, L left. Scale bars: 100 (a-d) and 50 (e) µm.

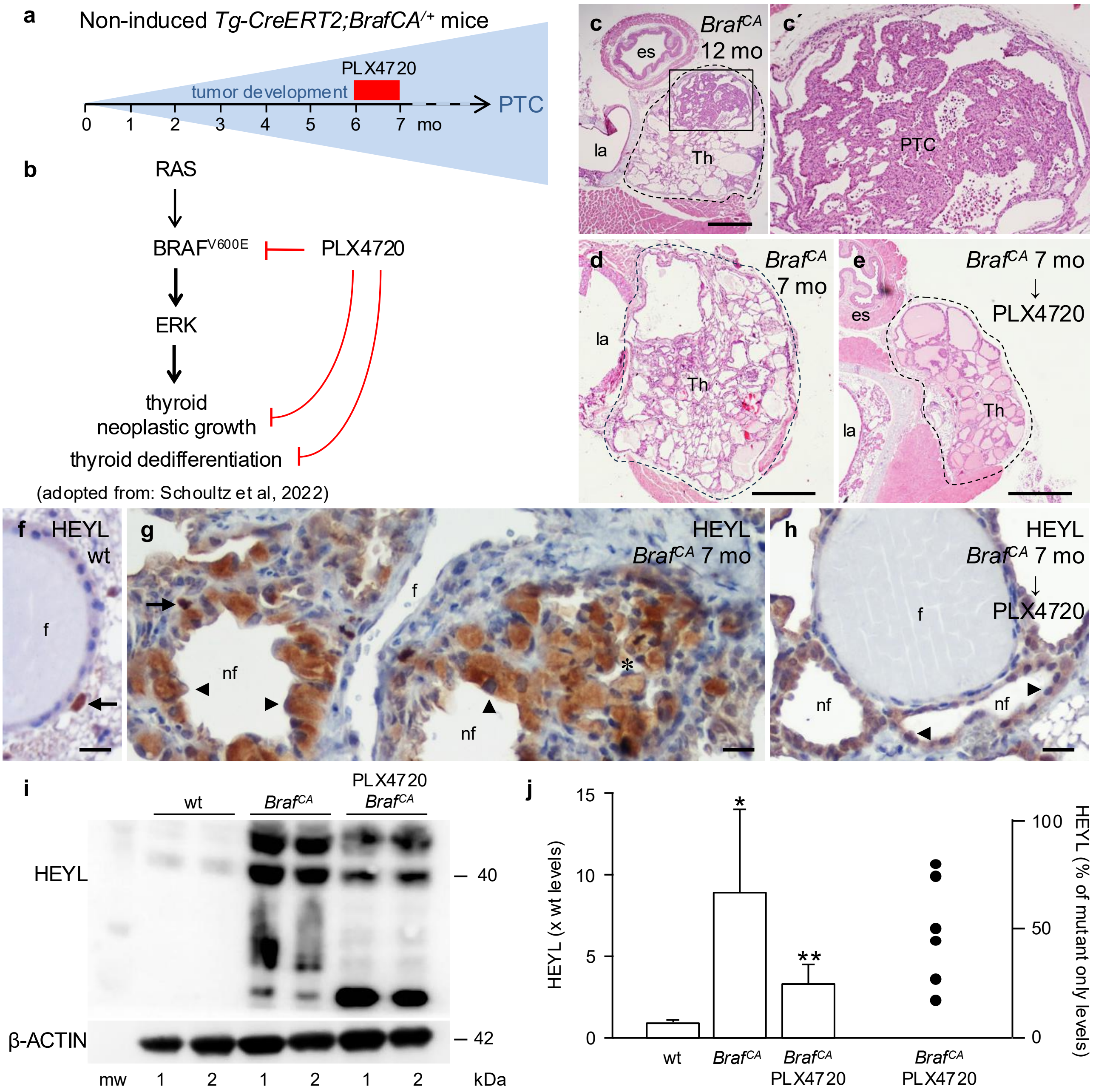

Supplementary Fig. 7. Upregulated Heyl expression in Braf mutant thyroid cells. A Braf mutant allele (Braf^{CA}) encoding BRAF^{V600E} was conditionally expressed under the thyroglobulin (Tg) promoter in *Tg-CreERT2;Braf$^{CA/+}$* mice. In this model, spontaneous Cre-mediated recombination (in absence of tamoxifen) leads to clonal development of papillary thyroid carcinoma (PTC), as previously described (Schoultz et al [46]). **a** Mouse PTC model and drug treatment protocol. **b** Principal effects of BRAF^{V600E} and mutant Braf kinase inhibition in the model. **c-e** Thyroid histology in *Braf^{CA}* mutant mice of different age without (c, d) or with (e) PLX4720 treatment. c´ shows high power of boxed area in (c). **f-h** HEYL immunohistochemsitry in thyroid of 7 months (mo) old wildtype (wt) and *Braf^{CA}* mice with/without PLX4720 treatment as outlined in (a). HEYL^{+} interstitial (arrows) and follicular (arrow-heads) cells are indicated. Asterisk in (g) indicates HEYL^{+} papillary microcarcinoma. Th thyroid lobe, la larynx, es esophagus, f follicle, nf, neoplastic follicle. Scale bars: 500 (c-e) and 25 (f-h) µm. **i, j** Western blot analysis of HEYL in duplicate samples (1, 2) from individual mice (n=3) derived from the same experiment with the gels/blots being processed in parallel; (j) shows mean±SD (n=6, double samples from 3 mice/group) with wt level set to ”1” (left panel) and individual values as compared to *Braf^{CA}* level set to 100% (right panel); *p=0.0242, **p=0.0254, both vs wt (t test, two-sided). Uncropped blot data are provided in end of this file. mw molecular weight ladder.

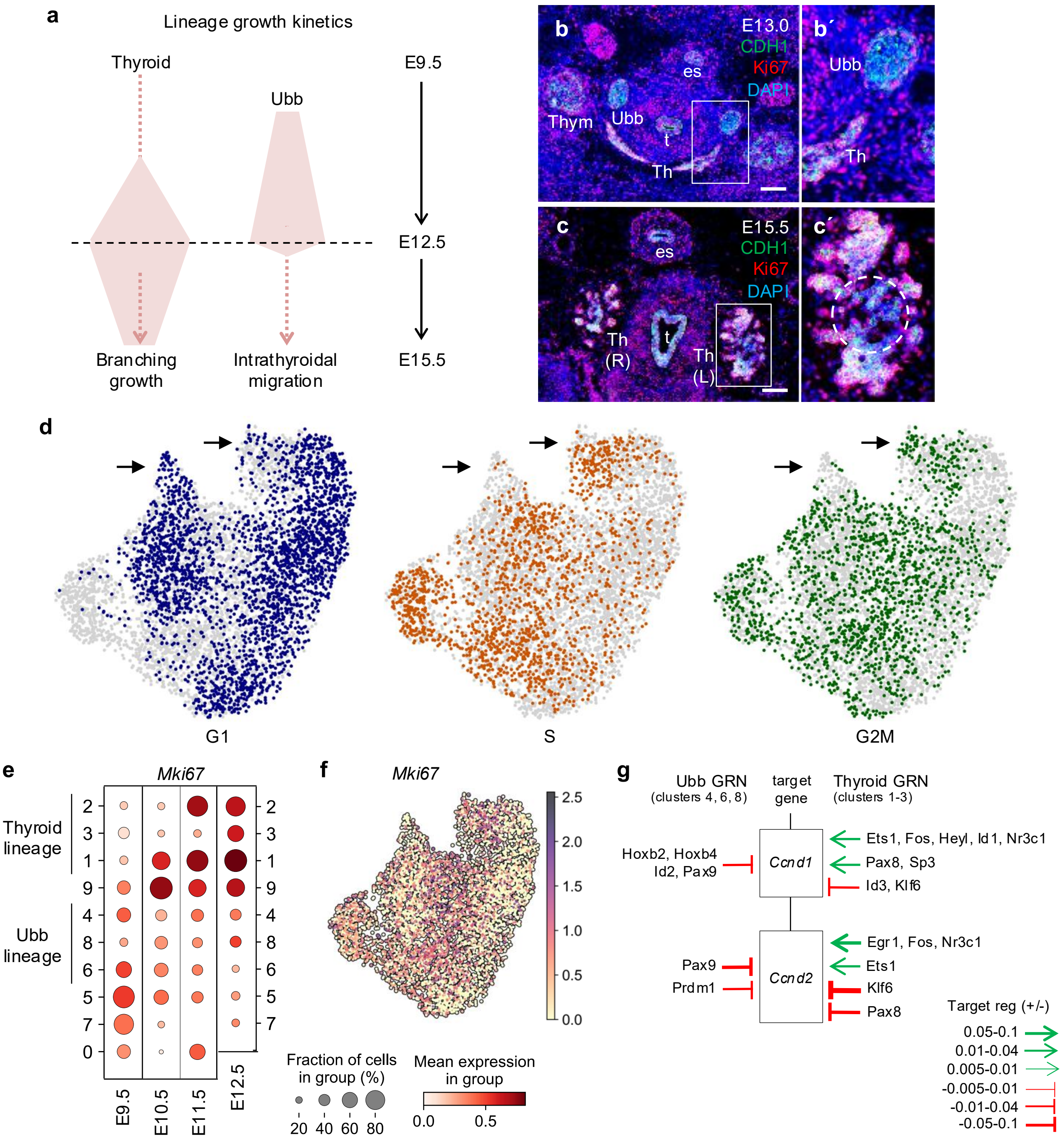

Supplementary Fig. 8. Different growth dynamics of thyroid and Ubb lineages during development. a Depicted differences in thyroid and Ubb cell proliferation across embryonic day (Eday) based on Ki67 expression patterns, as previously reported [15, 53]. **b, c** Ki67 expression before (b) and after (c) the paired Ubb merged with the midline thyroid bilaterally. Double immunofluorescence of Ki67 and CDH1/E-cadherin with DAPI nuclear staining. Boxed areas are shown at high power in (b´, c´) with Ubb rudiment encircled in (c´). Th thyroid (R, L right and left lobes), Ubb ultimobranchial body, t trachea, es esophagus, Thym thymus. Scale bars: 100 µm. **d** UMAP embeddings with cells colored according to whether they are predicted to be in cell cycle phase G1 (blue, left), S(orange, center) or G2M (green, right). Cells in each plot are colored gray if they are not in the corresponding phase. **e** Dot plots of cluster-specific *Mki67* expression across Eday. Dot size indicates the fraction of *Mik67*$^{+}$ cells whereas color indicates the mean log2-normalized expression. **f** UMAP embedding of *Mki67*. Scalebar indicates log2-normalized expression. **g** Predicted transcriptional regulation of *Ccnd1* and *Ccnd2* encoding cyclin D in Ubb and thyroid *in silico* GRNs identified by CellOracle. Magnitude of predicted up- and downregulation are indicated by sharp (green) or blunt (red) arrows and arrow thickness representing mean cluster-specific TF-target gene scores. Source data are provided in Supplementary Data 5.

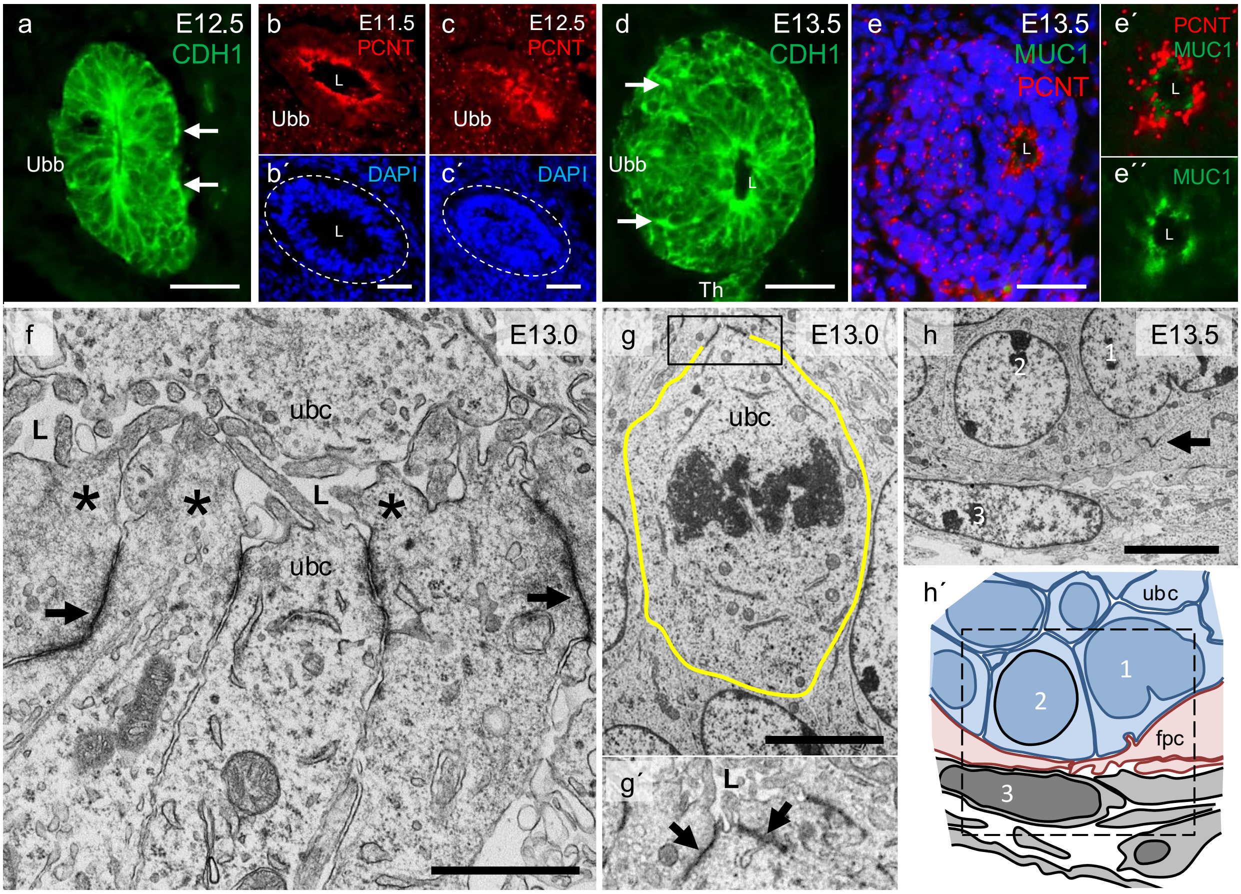

Supplementary Fig. 9. Morphodynamic changes of the developing Ubb. Immunofluorescence and transmission electron microscopy of wildtype ultimobranchial bodies (Ubb) between E11.5 and E13.5. **a-e** Lumen involution accompanying epithelial multilayering. Redistribution of CDH1/E-cadherin (a, d), PCNT/pericentrin (b-c´) and MUC1/mucin-1 (e-e´´). Arrows indicate basally located CDH1^{+} foci. **f** Apical constriction of Ubb cells. Arrows indicate elongated adherens junctions; asterisks mark protrusions of apical cytoplasm. **g** Ubb cell (outlined) undergoing oriented cell division signified by mitotic spindle perpendicular to the apical-basal axis. **g´** High power of boxed area in (g) with a much narrowed apical surface of the same mitotic cell. **h** Peripheral portion of Ubb partly invested by cells derived from the midline thyroid primordium. Arrows indicate focal adhesions established between the two cell types. **h´** Cartoon reproducing the motif in (h) (framed) with the deduced cell identities in *blue*: Ubb cells; in *pink*: thyroid cells; in *gray*: mesenchymal cells. Some corresponding cells are numbered 1-3 for clarity. L lumen, ubc ultimobranchial body cell, fpc folliclar progenitor cell. Scale bars: 50 (a-e), 5 (g, h) and 2 (f) µm.

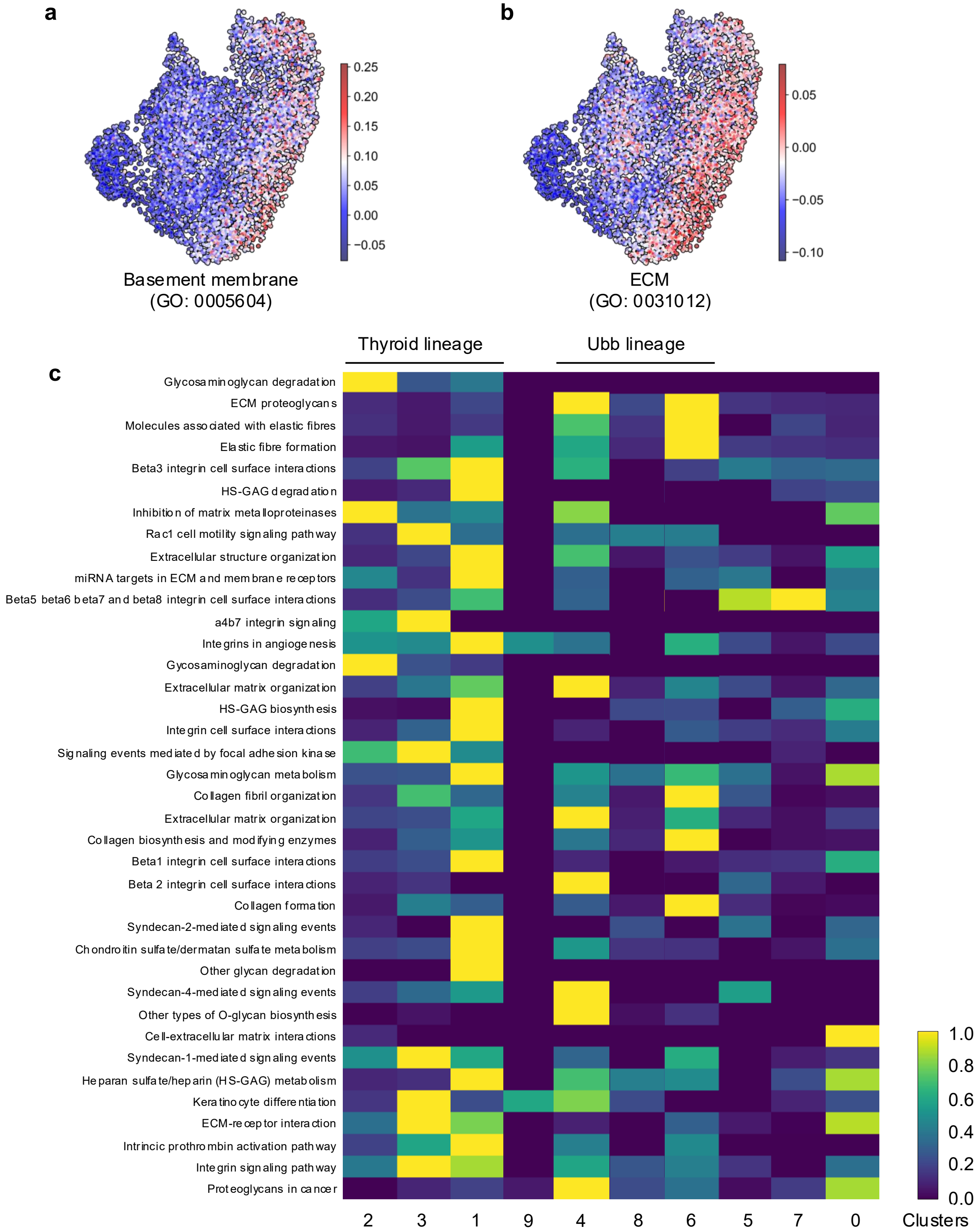

Supplementary Fig. 10. Differential expression of genes involved in turnover and regulation of extracellular matrix constituents during thyroid and ultimobranchial lineage development. a, b UMAPs displaying the per cell scores of basement membrane (a) and extracellular matrix (b) genes collectively identified obtained from the Gene Ontology database. Scores are defined as the average log2-normalized expression of the set of genes minus the average log2-normalized expression of a background set. Ubb, ultimobranchial body. Scale bar indicates gene set score. **c** Heatmap of a curated list of pathways from Gene Ontology, Reactome and WikiPathway databases associated with extracellular matrix (ECM) with heat indicating the row normalized −log10 adjusted p-values (Benjamini–Hochberg) obtained from an enrichment analysis (with EnrichR) of the genes differentially upregulated (adjusted p-value < 0.01, log2-fold change > 1.0) in each cluster over a background comprising the remaining clusters. Scale bar indicates scaled –log10 (adjusted value). Supplementary to Fig. 5. Source data are provided in Supplementary Data 1.

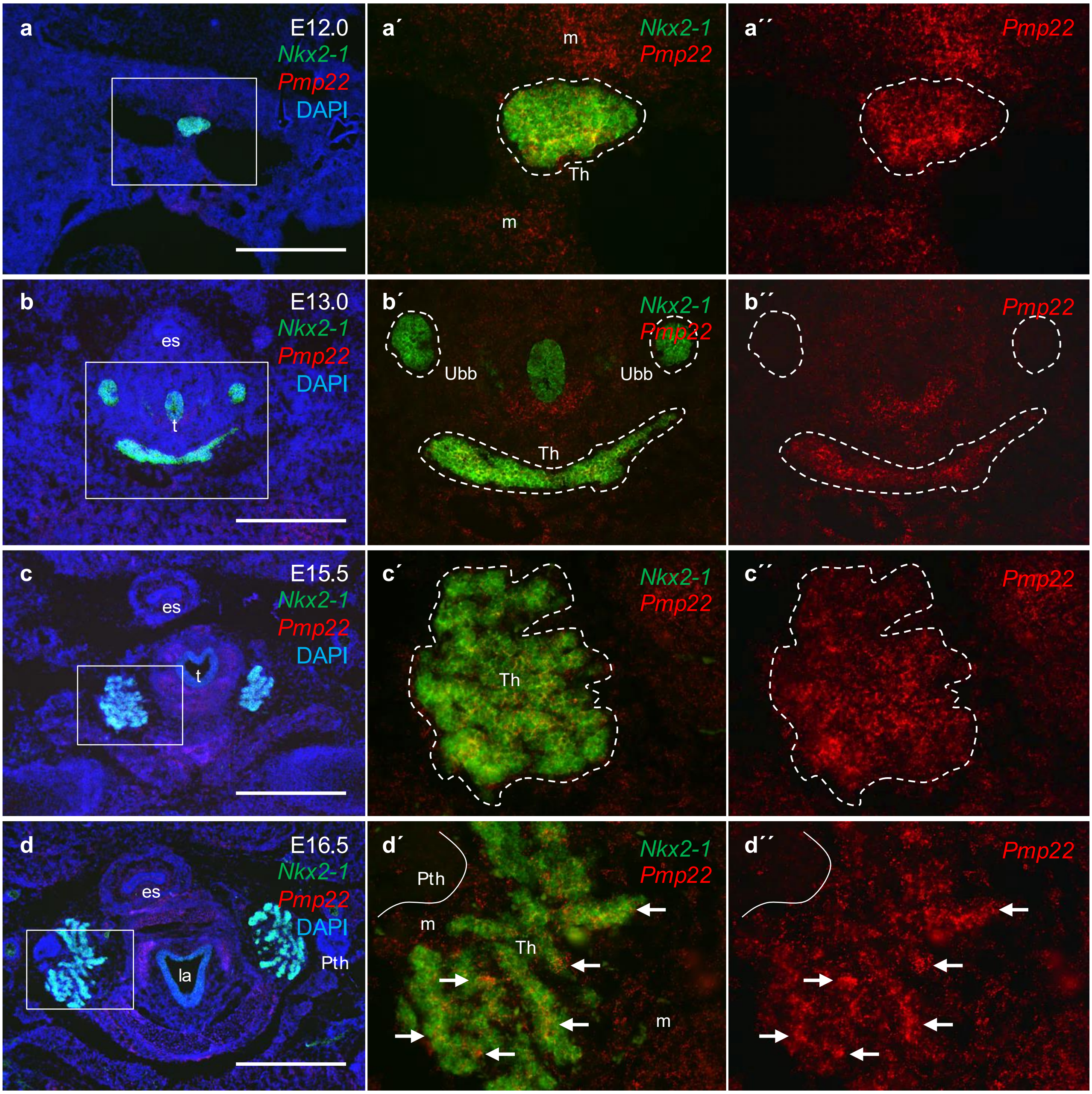

Supplementary Fig. 11. Pmp22 expression in the developing mouse thyroid. RNAscope Multiplex fluorescent analysis of *Pmp22* vs *Nkx2-1*. Middle and right panels show high power of the corresponding boxed areas to the left, without DAPI nuclear stain or with red channel only for improved clarity. **a** Midline thyroid primordium at E12.0. **b** Thyroid primordium growing bilaterally at E13.0. **c** Prospective thyroid lobes at E15.5. **d** Thyroid lobes undergoing branching growth at E16.5. Thyroid and ultimobranchial bodies are encircled, parathyroid perimeter outlined. Arrows indicate *Pmp22*+ branching parenchyma. Th thyroid, Pth parathyroid, Ubb ultimobranchial body, t trachea, la larynx, m mesenchyme. Scale bars: 500 µm.

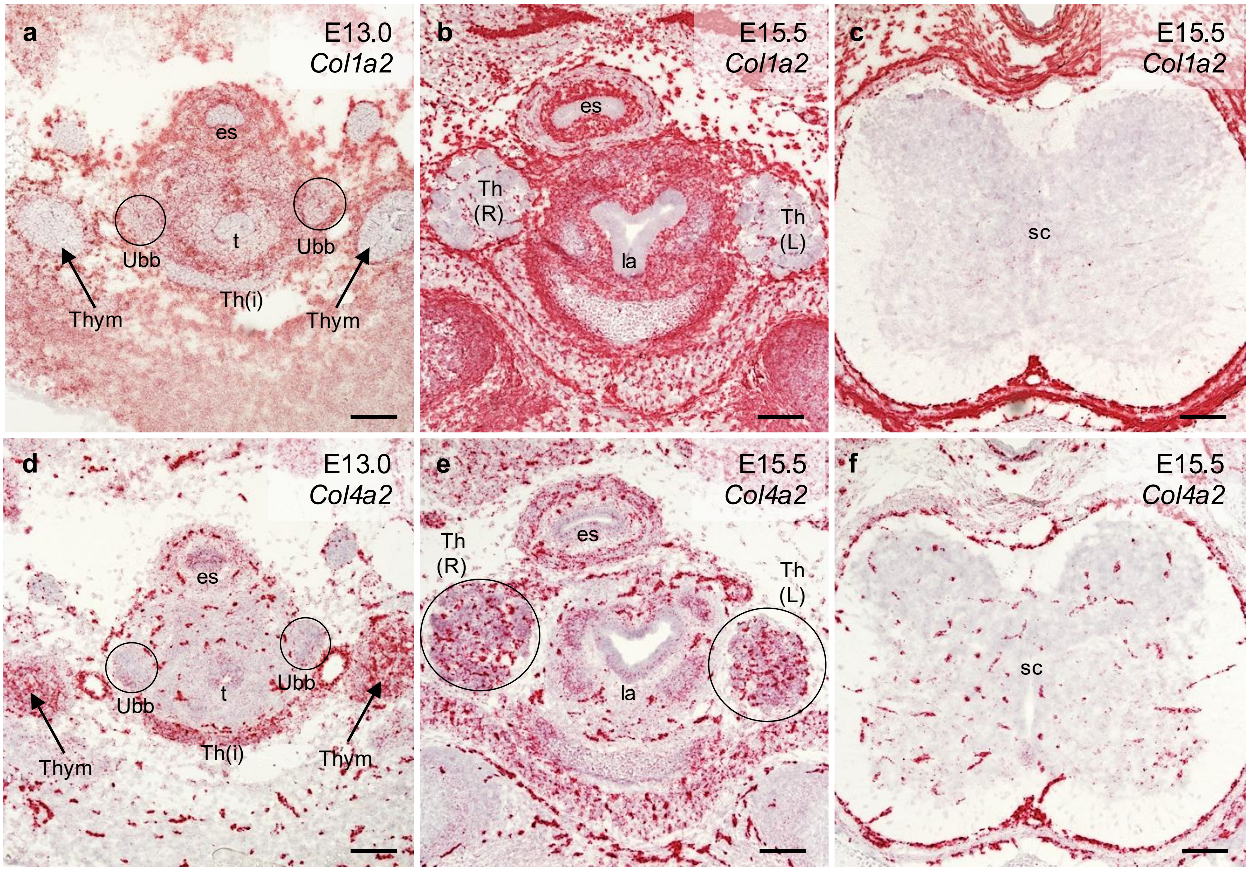

Supplementary Fig. 12. Differential expression of collagens type I and IV in embryonic neck tissues. Overview RNAscope images of *Col1a2* (**a-c**) and *Col4a2* (**d-f**) mRNA expression supplementary to Fig. 5c-f. Probes were hybridized onto parallel transverse sections obtained from paraformaldehyde-fixed/frozen E13.0 and E15.5 embryos. Ubb is encircled in (a, d) and right (R) and left (L) thyroid lobes are encircled in (e) för clarity. For comparison, collagen I-negative/collagen IV-positive microvessels are visualized in nervous tissue in (c, f). Th thyroid, Th(i) presumptive isthmus portion of thyroid, Ubb ultimobranchial body, Thym thymus, la larynx, t trachea, es esophagus, sc spinal cord. Scale bars: 100 µm.

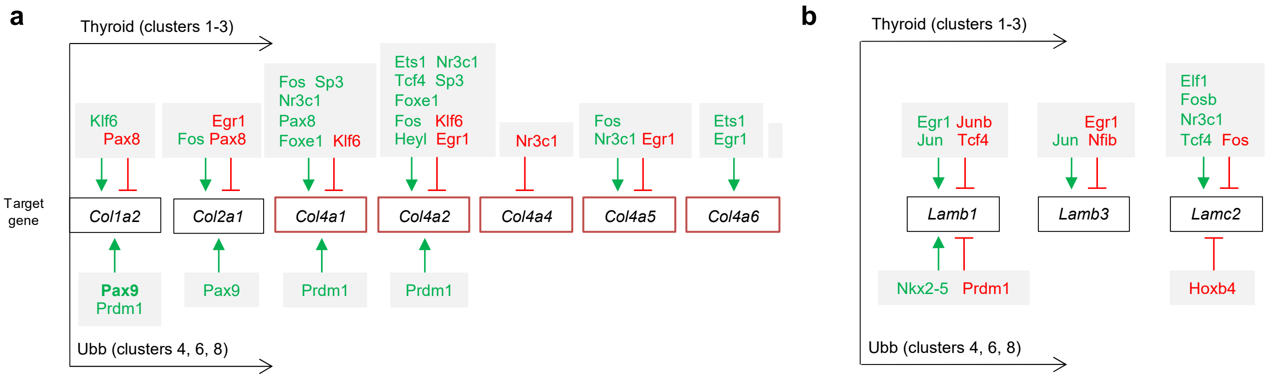

Supplementary Fig. 13. Inferred regulatory networks of collagen and laminin genes differ between thyroid and Ubb lineage cells. Schematic subnetworks comprising collagen (*Col*) I and IV genes (**a**) and laminin (*Lam*) genes (**b**) identified by CellOracle in clusters with thyroid and ultimobranchial lineage identities. Genes encoding basement membrane-specific collagen type IV alpha-chains are boxed in red. Predicted up- versus downregulation by the indicated transcription factors (TFs) are colored green or red and by corresponding sharp or blunt arrows. No information is provided on strength of betweenness designating importance of a single TF among others in a given subnetwork. Supplementary to Fig. 5 and 6. Source data are provided in Supplementary Data 5.

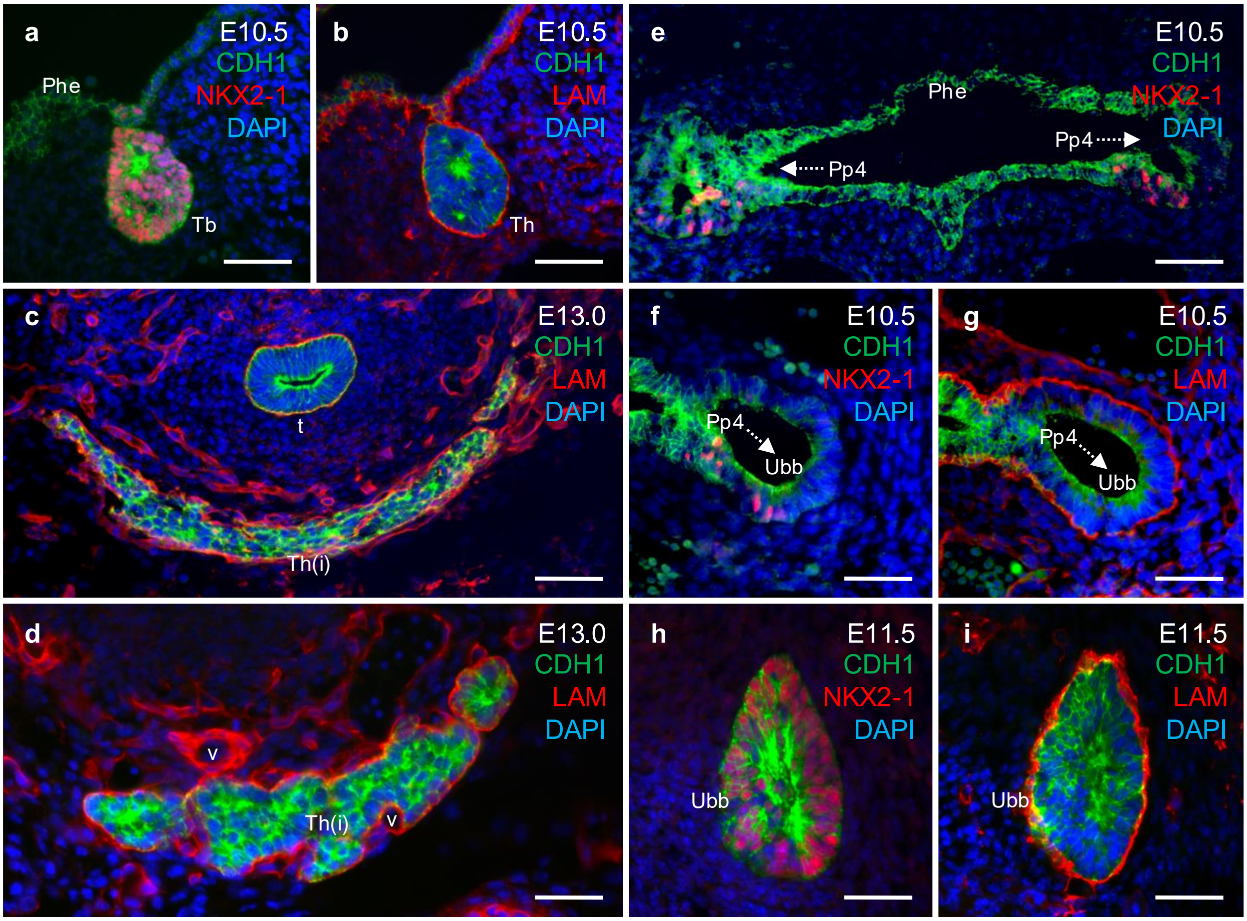

Supplementary Fig. 14. Laminin in basement membranes of the developing thyroid and ultimobranchial bodies. Pairwise double immunofluorescence of NKX2-1 versus laminin (LAM) and CDH1/E-cadherin with DAPI nuclear staining for identification of early primordial stages (on parallel sections in: a, b, f, g, h and i). Supplementary to Fig. 6. **a, b** Thyroid bud. **c, d** Presumptive thyroid isthmus. **e-g** Pharyngeal pouch; arrows indicate pouch-Ubb transition. **h, i** Ultimobranchial body. Phe pharyngeal endoderm, Tb thyroid bud, Th(i) isthmus portion of thyroid, Pp4 fourth pharyngeal pouch, Ubb ultimobranchial body, v microvessel. Scale bars: 100 (a-c, e) and 50 (d, f-i) µm.

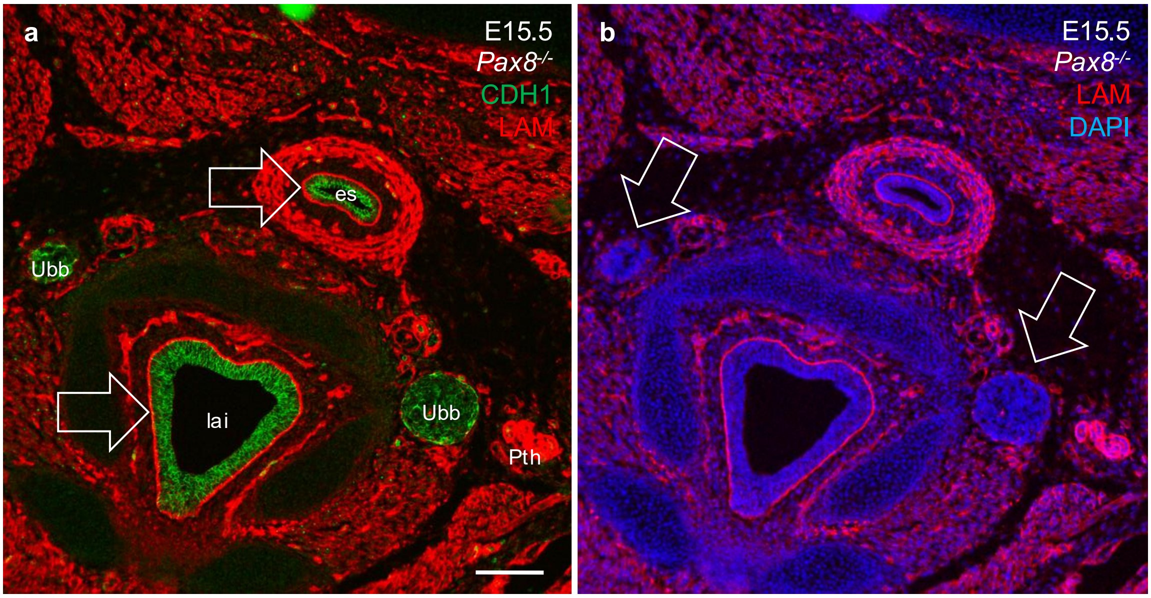

Supplementary Fig. 15. Selective loss of the Ubb laminin envelope in *Pax8* null mice. Overview of laminin (LAM) distribution in cross section of anatomical structures in the neck in mutant embryos lacking a thyroid gland. Supplementary to Fig. 6o. **a, b** Identical motifs with co-staining of CDH1/E-cadherin (a) and DAPI (b) for improved visualization of different laminin expression levels among organs. Arrows in (a) indicate LAM^+ basement membrane surrounding the esophageal and laryngeal epithelium, respectively. Arrows in (b) indicate discontinuous basement membrane with poor laminin content in Ubb. Pth parathyroid, Ubb ultimobranchial body, es esophagus, lai larynx interior. Scale bar: 100 µm.

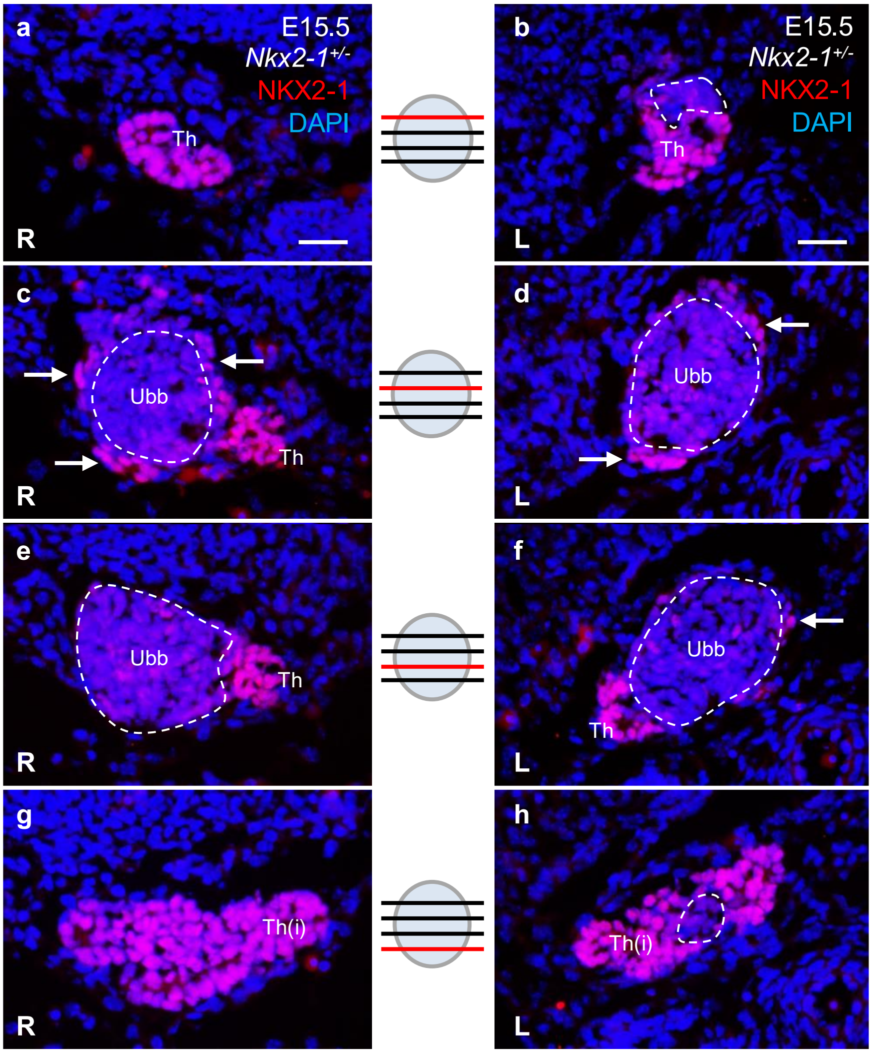

Supplementary Fig. 16. Impaired thyroid developmental growth in *Nkx2.1* heterozygous knockout mice. a-h Stacks of NKX2-1 immunofluorescent images from serially sectioned thyroid lobe rudiments obtained at E15.5; corresponding section levels of the two lobes are indicated in centre. Supplementary to Fig. 6p. Residual NKX2-1^{low} cells of the ultimobranchial body (encircled) are incompletely surrounded by NKX2-1^{high} cells (arrows) derived from the thyroid primordium. Lateral ends of the prospective thyroid isthmus are indicated in bottom images. Counterstaining with DAPI identifies all nucleated cells. Th thyroid, (i) isthmus, Ubb ultimobranchial body, R right side, L left side. Scale bar: 25 µm.

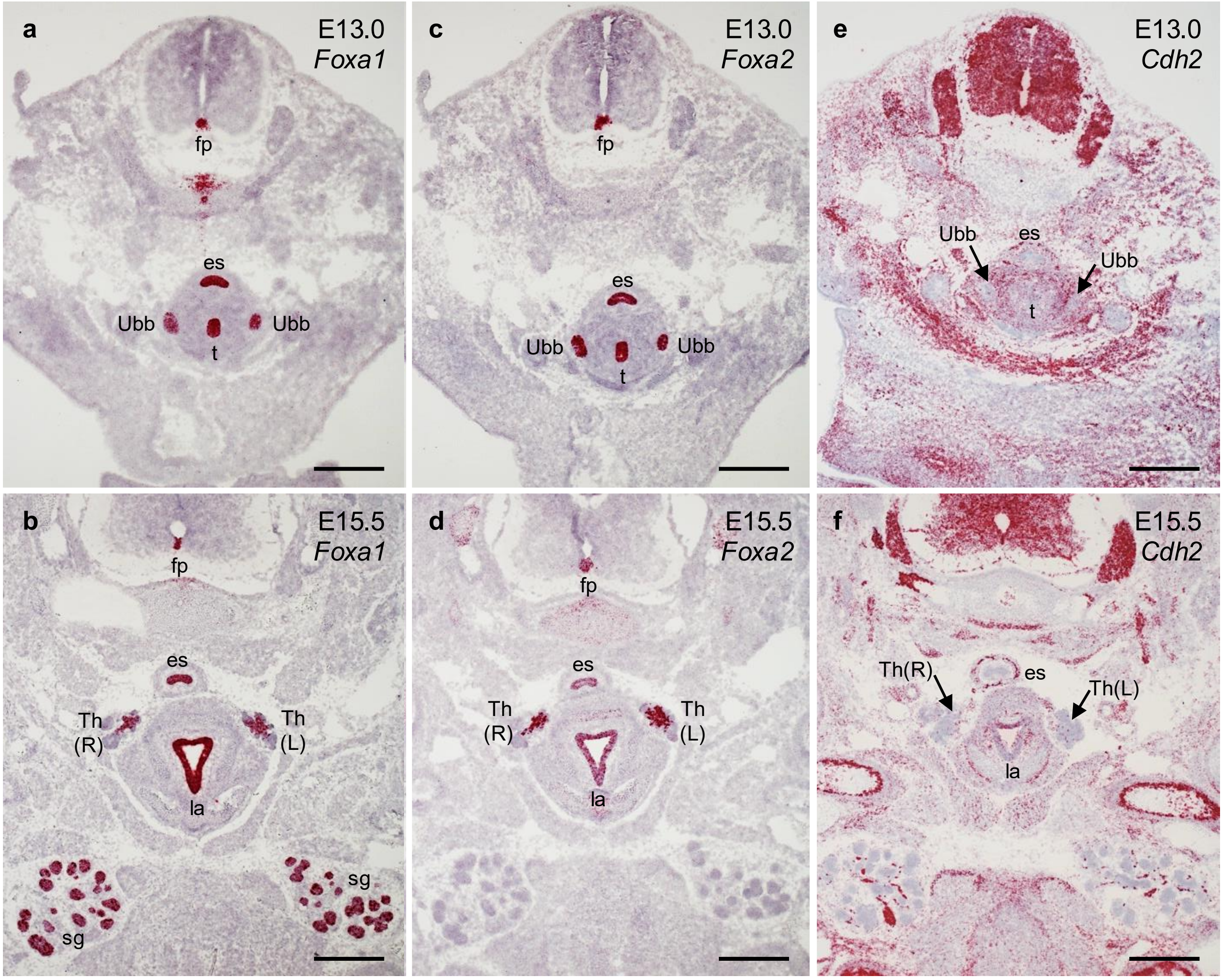

Supplementary Fig. 17. Expression pattern of Foxa1, Foxa2 and N-cadherin/Cdh2 in embryonic tissues. Overview RNAscope images of *Foxa1* (**a, b**), *Foxa2* (**c, d**) and *Cdh2* (**e, f**) supplementary to Figs. 7 and 8. Probes were hybridized onto parallel transverse sections obtained from paraformaldehyde-fixed/frozen E13.0 and E15.5 embryos. Fp floor plate, Th thyroid (R, L right and left lobes), Ubb ultimobranchial body, la larynx, es esophagus, sg salivary gland. Scale bars: 500 µm.

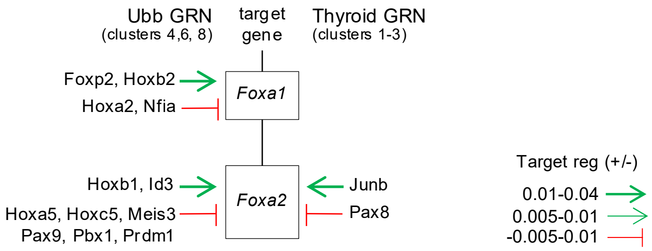

Supplementary Fig. 18. Transcriptional regulation of *Foxa1* and *Foxa2* in Ubb and thyroid gene regulatory networks as identified by CellOracle. Predicted up- and downregulation are indicated by sharp (green) or blunt (red) arrows and arrow thickness representing mean cluster-specific GRN TF–target gene interaction scores. Supplementary to Fig. 7g-u. Source data are provided in Supplementary Data 5.

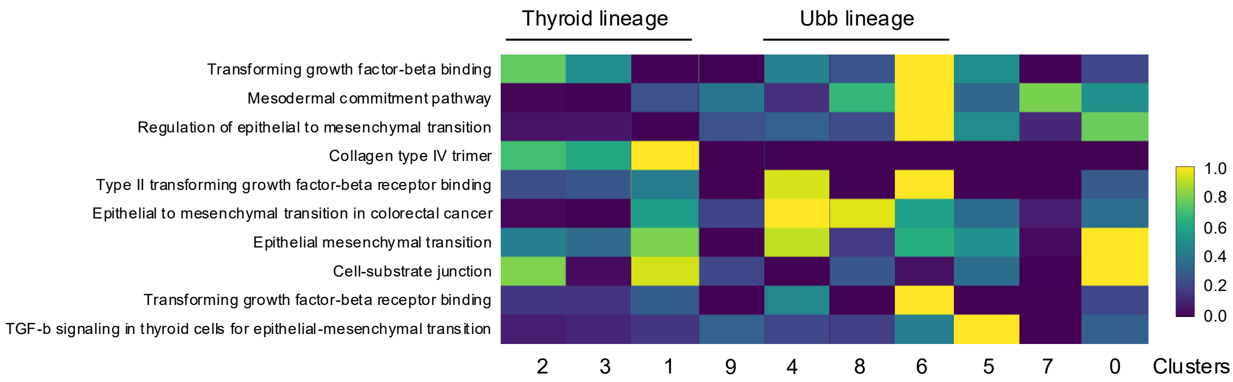

Supplementary Fig. 19. Differential expression of genes implicated in epithelial-to-mesenchymal transition thyroid and Ubb lineage cells. Heatmap of a curated list of pathways associated with epithelial-mesenchymal transition (EMT) with heat indicating the row normalized −log10 adjusted p-values (Benjamini–Hochberg). Corresponding cluster numbers and colors are indicated. Ontology terms obtained from Gene Ontology and WikiPathway databases. Supplementary to Fig. 8h-j.

Supplementary Table 1. Predicted Nkx2-1 target genes identified in the gene regulatory network derived from clusters 1-3 corresponding to thyroid lineage cells.

Nkx2-1 target genes		Predicted co-regulation by other thyroid-TFs	
Upregulated (n=33)[1]		Upregulated by:	Downregulated by:
	coef_mean		
Rdh10	0.029471	Klf6, Nfe2l2, Nr3c1, Pax8	Fos
Tcf4	0.025189	Batf3, Fos, Fosb, Heyl, Jun, Nr3c1, Pax8	Crem, Klf6
Grip2	0.019240	Egr1, Foxe1, Heyl, Sp3	Klf6
Stt3a	0.017647	Egr1, Pax8	Foxe1, Heyl, Sox9, Sp3
Fundc2	0.015592	Egr1	-
Pet100	0.014023	Arntl, Egr1, Elk1, Ets1, Heyl, Id1, Junb	Crem, Fos, Fosb, Id3, Klf6, Nfe2l2, Nr3c1
Foxp1	0.013232	Fos, Foxe1, Lef1, Nfib, Nr4a2, Sp3	Heyl, Pax8
Edc3	0.013165	Klf6	Creb5, Fosb, Heyl, Junb
Pygm	0.012870	Klf6	Ets1, Sp3
Pax8	0.012797	Creb5, Crem, Ets1, Fos, Foxe1, Heyl, Hivep3, Id3, Maf, Nfic, Rorc, Tcf4	Fosb, Nfib
Hpcal1	0.011732	Ets1, Klf6, Nr3c1, Pax8, Sp3	Egr1
Tmem213	0.011022	Sp3	Heyl, Id1
Fance	0.010977	Egr1, Elf3	Pax8
S100a10	0.010839	Ets1, Klf6, Nr3c1	Sp3
Hoxb2	0.010095	Bcl11b, Ets1, Id1, Nr4a2, Sp3	Egr1, Fos, Klf6, Nr3c1, Tcf4
Luzp2	0.008979	-	-
Stxbp6	0.008855	Egr1, Ets1, Heyl	Elf3, Klf6, Nfib
Scaper	0.007441	-	-
Aars2	0.007268	-	-
Atp5d	0.006988	Cux1, Egr1, Heyl, Id1, Id3, Nfe2l2, Pax8	Bach1, Fosb, Foxe1
Mypop	0.006807	-	-
Kcmf1	0.006807	Egr1, Heyl, Id1	Id3
Lmo4	0.006045	Bcl11b, Egr1, Id1, Isl1, Klf6, Maf, Nr3c1	Heyl, Pax8
Kctd12b	0.005887	-	-
Mapk1ip1	0.005732	Egr1, Klf6	Elf3, Fos, Sp3
Ubash3b	0.005693	Cux1, Egr1, Ets1, Jun, Nr3c1, Sp3	Foxe1, Klf6
Flrt1	0.005598	-	-
Greb1l	0.005409	Ets1, Fos, Sp3, Tcf4	Klf6
Snx14	0.005401	Heyl, Jun, Nr3c1	Fos
Faap100	0.005353	-	-
Cyp39a1	0.005300	Klf6	Egr1, Fos
Sestd1	0.005062	Egr1	-
Invs	0.005006	Id3	Heyl
Downregulated (n=18)[2]		Upregulated by:	Downregulated by:
Cadm1	-0.028725	Egr1, Sp3	Fos, Nr3c1
Hdhd2	-0.017271	Egr1, Elf3, Heyl, Sp3	-
Igfbp2	-0.016711	Fos, Isl1, Maf, Sp3	Bach1.Egr1. Heyl, Id1, Id3
Pole3	-0.013811	-	Nr3c1
Ugcrh	-0.012875	Elf3	-
Hspd1	-0.012783	Egr1	Bcl11b, Creb5, Fos, Heyl, Id3, Junb, Klf6 Nr3c1, Nr4a2, Sp3
Ccar2	-0.012654	Elk3, Ets1	Klf6
Sdhd	-0.011841	Egr1	Klf6, Nr3c1, Nr4a2
Snrpd2	-0.011760	Egr1, Elf3, Elk3	Fos, Klf6
Pno1	-0.009733	Egr1	Arntl, Id3
Gnas	-0.008980	Arntl, Egr1, Elf3, Ets3, Heyl, Id3	Fos, Hivep3, Klf6, Maf, Sp3
Pnf2	-0.008870	Junb	Egr1, Heyl, Id3
Cldn9	-0.007990	-	Pax8
Orc6	-0.007936	Egr1, Ets1	Klf6, Sp3
Sept11	-0.007780	Hoxb2	Id3
Adprhl2	-0.007260	Ets1, Nfe2l2	-
Angpt2	-0.006513	Batf3, Jun, Isl1, Mecom, Pax8	Klf6, Nr3c1, Sp3
Exo5	-0.005435	Nr3c1	Egr1, Mecom

TF transcription factor
[1]listed from highest to lowest scores (coef_mean)
[2]listed from lowest to highest scores (negative coef_mean)
Source data are provided in Supplementary Data 5.

Supplementary Table 2. Predicted Nkx2-1 target genes identified in the gene regulatory network derived from clusters 4, 6 and 8 corresponding to the Ubb/C-cell lineage.

Nkx2-1 target genes		Predicted co-regulation by other Ubb-TFs	
Upregulated (n=35)[1]		Upregulated by:	Downregulated by:
	coef_mean		
Pfn2	0.049089	Id2, Id3	Nkx2-5
Igfbp2	0.032229	Id2	Id3, Prdm1
Hpcal1	0.031613	-	Meis2, Prdm1
Nrp2	0.021344	Pax9	Id2, Id3, Prdm1
Cspp1	0.020172	Irx2	-
Pno1	0.019892	Id2	-
Foxp1	0.018758	Foxp2, Id3, Nfia, Nfib, Pax9	Hoxb2, Hoxb4, Hoxc5, Id2, Irx2, Prdm1
Stxbp6	0.016903	-	-
Fxyd7	0.016773	Pax9	-
Msrb2	0.015009	-	-
Flrt1	0.014910	Id2, Pbx1, Prdm1	Id3, Nfib, Nkx2-5
Faap100	0.013429	-	Nfia, Pax9
Cops5	0.013040	-	-
Upk1a	0.012801	Prdm1	-
St8sia6	0.011184	-	-
Magi1	0.010506	-	-
Pet100	0.009633	Nkx2-5	Id3, Prdm1
Rspo1	0.009595	-	-
Hoxb4	0.009513	Hoxa5, Hoxb1, Hoxb2, Hoxb5, Hoxc5 Meis1, Meis2, Prdm1, Zfhx3	Id2, Id3
Dbp	0.009280	-	-
Gstk1	0.008742	Prdm1	-
Nol10	0.007874	-	-
Med26	0.007475	-	-
Copz2	0.007414	Nkx2-5	-
Tmem213	0.006635	Zfhx3	Id2
Aars2	0.006408	-	-
Tnfrsf12a	0.005858	Pax8	-
Dctd	0.005678	-	Pax9
Kat5	0.005637	-	-
Timm23	0.005542	Id2	Pax9
Poc5	0.005315	-	-
Nefm	0.005279	Id2, Meis1, Nfib, Pbx1	Hoxb2, Hoxc5, Foxp2, Meis2, Nfia, Prdm1
Ptcd3	0.005201	Hoxa2, Hoxb6	Bach2
Tmem171	0.005153	Hoxb2	-
Med14	0.005080	-	Prdm1

Downregulated (n=53)[2]		Upregulated by:	Downregulated by:
Fgf8	-0.096228	Irx2, Prdm1	-
Cadm1	-0.044912	Prdm1	Nkx2-5
Hspd1	-0.044258	Id3	Nkx2-5, Prdm1
Uqcrh	-0.040713	-	Etv1, Prdm1
Orc6	-0.038694	Nkx2-5	-
Galk1	-0.035238	Id3, Nkx2-5	Bach2
Lmo4	-0.033411	Id2, Meis1, Prdm1	Hoxa2, Hoxb4, Hoxc5, Id3, Nkx2-5
Efna5	-0.026840	Hoxa2, Hoxb4	-
Lpar4	-0.024157	Prdm1	-
Rad54l	-0.022496	-	Nkx2-5
Trip13	-0.021666	-	Etv1
Sdhd	-0.020869	Pax9	Nkx2-5
Tada1	-0.018832	Pax9	-
Anxa2	-0.016896	Nfib, Nkx2-5	Prdm1
Foxg1	-0.016151	Id2	Id3, Nkx2-5, Prdm1
Pole3	-0.015971	-	-
Gtf3c6	-0.015043	Id3, Prdm1	-
Cited2	-0.014739	Id2, Id3, Prdm1	-
Hoxb6	-0.012521	Hoxb2, Hoxb4, Hoxc5, Prdm1	Id3, Meis2, Nkx2-5

Supplementary Table 2. continued

Nkx2-1 target genes		Predicted co-regulation by other Ubb-TFs	
Downregulated (n=53)[2]		Upregulated by:	Downregulated by:
	coef_mean		
Casd1	-0.011648	-	-
Atp5d	-0.011592	Meis1, Pax9	Id2, Prdm1
Edc3	-0.011537	-	-
Abcb8	-0.011396	Id3, Prdm1	-
Haus5	-0.011052	-	-
Tgif1	-0.010553	Bach2, Hoxb2, Id2, Meis1, Meis2, Nkx2-5, Sox4	Meis3, Nfib
Nexn	-0.010532	-	-
Hdhd2	-0.010479	Bach2, Id3	-
Capn10	-0.010371	Prdm1	-
Rps6ka3	-0.009940	-	Nkx2-5
Gas2	-0.009886	Hoxb6, Id2, Meis1, Mesi2, Mesi3, Pbx1	Hoxb2, Nkx2-5
Tmem65	-0.009833	Prdm1	-
Arl4a	-0.008867	-	-
Dera	-0.008383	-	Nfia, Nkx2-5
Prep	-0.008370	-	-
Smndc1	-0.008251	Pax9	Nkx2-5
Hoxb2	-0.007594	Hoxa5, Hoxb1, Hoxb4, Hoxb5, Hoxc5, Id3, Meis2, Nfib, Prdm1, Zfhx3	Nfia
Slc6a15	-0.007575	Meis1	-
Cog8	-0.007176	Nkx2-5, Prdm1	-
Dhx32	-0.007130	Bach2, Pax9, Prdm1	Meis2
Pgam2	-0.006947	-	-
Zmiz2	-0.006737	-	Nkx2-5
Rmi2	-0.006449	-	-
Sgk3	-0.006283	Id3	-
Slitrk4	-0.006168	-	-
Cnot3	-0.006048	Nfia, Pax8	-
Kcnj2	-0.006029	-	Id3
Mapk1ip1	-0.005805	Pax9	-
Pcca	-0.005648	-	-
Ubash3b	-0.005576	-	-
Pitx1	-0.005548	Id3	Hoxb2, Id2, Pbx1
Poir1a	-0.005503	-	-
Xab2	-0.005454	Prdm1	Etv1
Snx14	-0.005389	-	-

TF transcription factor

[1]listed from highest to lowest scores (coef_mean)

[2]listed from lowest to highest scores (negative coef_mean)

Source data are provided in Supplementary Data 5.

Supplementary Table 3. Nkx2-1 target genes shared by thyroid and Ubb/C-cell lineage cells.

Genes (n=25)	Thyroid lineage (clusters 1-3)	Ubb lineage (clusters 4, 6, 8)	Encoded protein / main function(s)
Aars2	↑	↑	Alanyl-tRNA synthetase 2 / mitochondrial
Atp5d	↑	↓↓	ATP synthase delta unit / mitochondrial
Cadm1	↓↓↓	↓↓↓↓↓	Cell adhesion molecule 1 / cell adhesion
Edc3	↑↑	↓↓	Enhancer of mRNA decapping protein 1 / transcription
Faap100	↑	↑↑	Fanconi anemia core complex protein / DNA repair
Flrt1	↑	↑↑	Fibronectin leucine rich transmembrane protein 1 / cell adhesion
Foxp1	↑↑	↑↑	Forkhead box P1 transcription factor / development
Hdhd2	↓↓	↓↓	Haloacid dehalogenase-like hydrolase domain containing protein 2 / metabolism
Hoxb2	↑↑	↓	Homeobox B2 transcription factor / development
Hpcal1	↑↑	↑↑↑↑	Hippocalcin-like protein 1 / calcium binding, ferroptosis, autophagy
Hspd1	↓↓	↓↓↓↓↓	Heat shock protein 60 / mitochondrial
Igfbp2	↓↓	↑↑↑↑	Insulin-like growth factor binding protein 2 / growth regulation
Lmo4	↑	↓↓↓↓	LIM domain only 4 / transcription, development
Mapk1ip1	↑	↓	MAPK scaffold protein 1 / growth regulation
Orc6	↓	↓↓↓↓	Origin recognition complex subunit 1 / DNA replication
Pet100	↑↑	↑↑	Cytochrome C oxidase chaperone / mitochondrial
Pnf2	↓	↑↑↑↑↑	Profilin 2 / actin-based cytoskeleton
Pole3	↓↓	↓↓	DNA polymerase epsilon catalytic subunit 1 / DNA replication
Pno1	↓	↑↑	Partner of NOB1 homolog / ribosomal assembly
Sdhd	↓↓	↓↓↓	Succinate dehydrogenase / mitochondrial
Snx14	↑	↓	Sorting nexin 14 / intracellular transport, autophagy
Stxbp6	↑	↑↑	Syntaxin-binding protein 6 / cell adhesion, exocytosis
Tmem213	↑↑	↑	Transmembrane protein 213 / membrane transport
Ubash3b	↑	↓	Ubiquitin-associated and SH3 domain-containing protein B / cell signaling
Uqcrh	↓↓	↓↓↓↓↓	Ubiquinol-Cytochrome C reductase hinge protein / mitochondrial

Green arrows corresponding to coef_mean: ↑ 0.005-0.01; ↑↑ 0.01-0.02; ↑↑↑ 0.02-0.03; ↑↑↑↑ 0.03-0.04; ↑↑↑↑↑ >0.04
Red arrows corresponding to negative coef_mean: ↓ -0.005-0.01; ↓↓ -0.01-0.02; ↓↓↓ -0.02-0.03; ↓↓↓↓ -0.03-0.04; ↓↓↓↓↓ -0.04-0.05
Source data are provided in Supplementary Data 5.

Supplementary Table 4. General information on tissue expression pattern and function of enriched genes, recovered by scRNAseq analysis, in differentiated (calcitonin-positive) and immature (calcitonin-negative) cells of the Ubb/C-cell lineage.

Calca pos vs *Calca* neg Ubb cells

Gene	Encoded protein	Development	Endocrine	Function(s)
Nnat	Neuronatin	neural fate, brain	pituitary, pancreatic beta-cells	calcium regulation
Mest	Mesoderm-specific transcript homolog	mesoderm	adrenal	EMT
Foxa2	Forkhead box A	endoderm, lung, liver, pancreas	thyroid C cells, pancreas	pioneer transcription factor
Btg2	B-cell translocation gene 2	neurogenesis	pancreatic beta-cells	cell cycle
Clpx2	Complexin 2 (synaphin)	-	neuroendocrine	exocytosis
Ascl1	Achaete-scute homolog 1 (MASH1)	neurogenesis	adrenal, thyroid C cells, MTC	pioneer transcription factor
Cdc25b	Cdc25 isoform	early embryogenesis	MTC	cell cycle
Socs2	Suppressor of cytokine signaling 2	neuronal differentiation	pancreatic beta-cells	JAK/STAT signaling
Hair1	Hoxa adjacent long noncoding RNA 1	ESC lineage differentiation	-	RA regulation
Sst	Somatostatin	neuroendocrine differentiation	neuroendocrine, MTC	peptide hormone
Krt7	Keratin 7	epithelial differentiation	neuroendocrine	cytoskeleton
Prox1	Prospero homeobox 1	cell fate determination	neuroendocrine, thyroid C cells, MTC	EMT, secretory pathway

*Calca*_neg vs *Calca*_pos Ubb cells

Gene	Encoded protein	Function(s)
Hmcn1	Hemicentin 1 (HMCN1)	ECM, basement membrane organization, cell anchorage
Plagl1	PLAGL1 (ZAC-1)	transcription factor, ECM regulation, anti-proliferative
Hs6st2	Heparan sulfate 6 sulfotransferase 2	ECM regulation, morphogen/growth factor activation
Rbm12b2	RNA binding motif protein 12	post-transcriptional regulation
Smtnl2	Smoothelin-like 2	apical actin turnover
Dscc1	DNA replication and sister chromatid cohesion 1	DNA replication

---

List of genes related to data as presented in Main Figure 7e and f, respectively, based onscRNAseq analysis of E12.5 cells confined to Ubb clusters 4, 6 and 8. Gene/encoded protein characteristics are summarized from relevant literature on PubMed.
Ubb ultimobranchial body, MTC medullary thyroid carcinoma, EMT epithelial-mesenchymal transition, ECM extracellular matrix
Source data are provided in Supplementary Data 7.

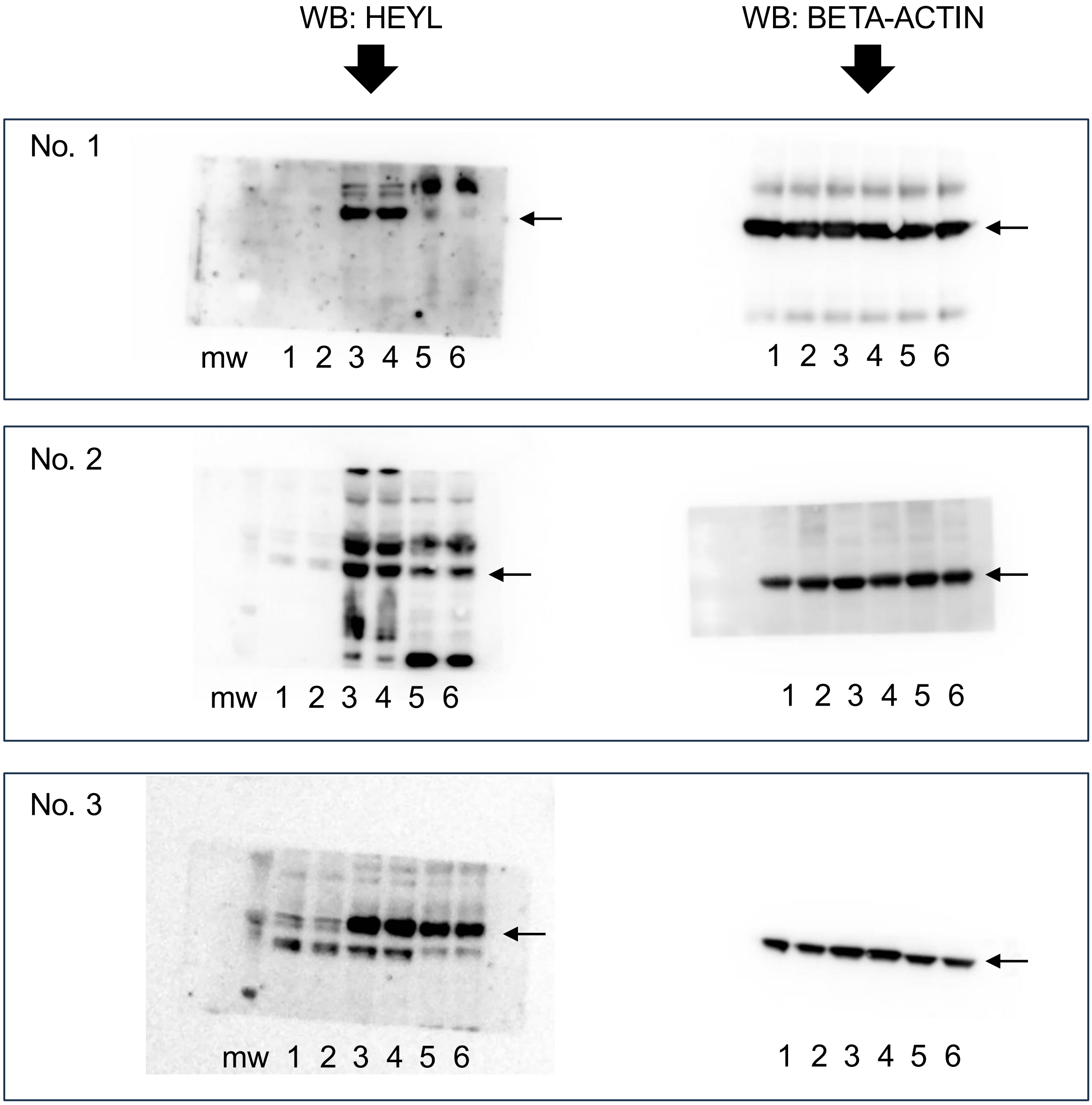

Uncropped scans of all blots related to Supplementary Figure 7. Western blot analysis of Heyl expression in duplicates from adult mouse thyroid tissues (n=3): wildtype (lanes 1+2), *Tg-CreERT2;Braf*$^{CA/+}$ (lanes 3+4) and PLX4720-treated *Tg-CreERT2;Braf*$^{CA/+}$ (lanes 5+6). Left-most lanes show molecular weight (mw) ladder. Arrows indicate bands with the expected molecular weight for HEYL (40 kDa) and beta-ACTIN (42 kDa), respectively. Immunoblots in Braf mutant thyroids reveal additional multiple bands likely reflecting differentially processed HEYL native protein, variably present in without/with Braf kinase inhibitor treatment.
