# Supplementary material for: Resolving thyroid lineage cell trajectories merging into a dual endocrine gland in mammals
Source: Nat Commun. 2026 May 25;17:6811. doi: 10.1038/s41467-026-73385-6 (PMC13385830; doi:10.1038/s41467-026-73385-6)
Supplement: Supplementary file 2 — Description of Additional Supplementary Files [file 41467_2026_73385_MOESM2_ESM.pdf]

Description of Additional Supplementary Files

File Name: Supplementary Data 1

Description: Single cell transcriptome analyses of the developing mouse thyroid and ultimobronchial body (Ubb).

Tables showing the cluster proportions at each embryonic day (Fig. 1k) and the differentially upregulated genes in clusters 0-9 (Fig. 1i) (log2foldchange > 1.0, adjusted p-value < 0.01).

File Name: Supplementary Data 2

Description: Predicted thyroid lineage drivers.

Table showing the genes predicted to be driving the thyroid lineage ranked according to their correlation with predicted thyroid fate probabilities (Fig. 2c, red). The columns indicate correlation of the gene expression with the per-cell thyroid fate probability, the associated p-value, multiple-testing corrected q-value, and low and high bounds of the correlation confidence interval.

File Name: Supplementary Data 3

Description: Predicted Ubb lineage drivers.

Table showing the genes predicted to be driving the Ubb lineage ranked according to their correlation with predicted Ubb fate probabilities (Fig. 2c, purple). The columns indicate correlation of the gene expression with the per-cell Ubb fate probability, the associated p-value, multiple-testing corrected q-value, and low and high bounds of the correlation confidence interval.

File Name: Supplementary Data 4

Description: Genes enriched in Heyl-expressing versus Heyl negative thyroid lineage cells.

Table showing genes significantly differentially upregulated (log2-foldchange > 1, adjusted p-value < 0.05) in Heyl+ (150 cells) compared to Heyl- (882 cells) in the thyroid lineage (clusters 1, 2, and 3). Heyl+ cells were defined as those with scran normalized expression of *Heyl* > 0 and Heyl- cells were those with MAGIC imputed expression of *Heyl* < 0.02 (cutoff defined at the lowest mode of the imputed expression distribution). Used in Fig. 3 (panel g).

File Name: Supplementary Data 5

Description: Thyroid and Ubb gene regulatory networks (GRNs).

Tables showing the filtered Ubb and Thryoid GRNs as inferred by CellOracle. Used in Fig. 2-4, 7-8.

File Name: Supplementary Data 6

Description: Morphometric analysis and Wester blot quantification.

Numerical values for graphs in Fig. 6 (panels e, f, g, q, r) and Supplementary Fig. 7 (panel j).

File Name: Supplementary Data 7

Description: Genes enriched in Calca-expressing versus Calca negative cells in the Ubb lineage.

Tables showing genes significantly differentially upregulated (log2-foldchange > 1, adjusted p-value < 0.05) in *Calca*$^{+}$ compared to *Calca*$^{-}$ cells and vice versa in the Ubb lineage comprising clusters 4, 6 and 8 at E12.5. *Calca*$^{+}$ cells are defined as those having normalized expression of *Calca* >0. Used in Fig. 7 (panels e, f) and associated with Supplementary Table 4.

File Name: Supplementary Data 8

Description: Marker gene expression in TT cells.

Uncropped and unprocessed Western blot images of: Calcitonin, E-cadherin/Cdh1, N-cadherin/Cdh2, Foxa2 and beta-actin. Doublet samples (1, 2) were analyzed. Molecular weight (mw) ladder in color is included in all blots. Arrows indicate bands with the expected molecular weight for each marker protein. Used in Fig. 9 (panel c).
