# Supplementary material for: Resolving thyroid lineage cell trajectories merging into a dual endocrine gland in mammals
Source: Nat Commun. 2026 May 25;17:6811. doi: 10.1038/s41467-026-73385-6 (PMC13385830; doi:10.1038/s41467-026-73385-6)
Supplement: Supplementary file 10 — Supplementary Data 8 [file 41467_2026_73385_MOESM10_ESM.pdf]

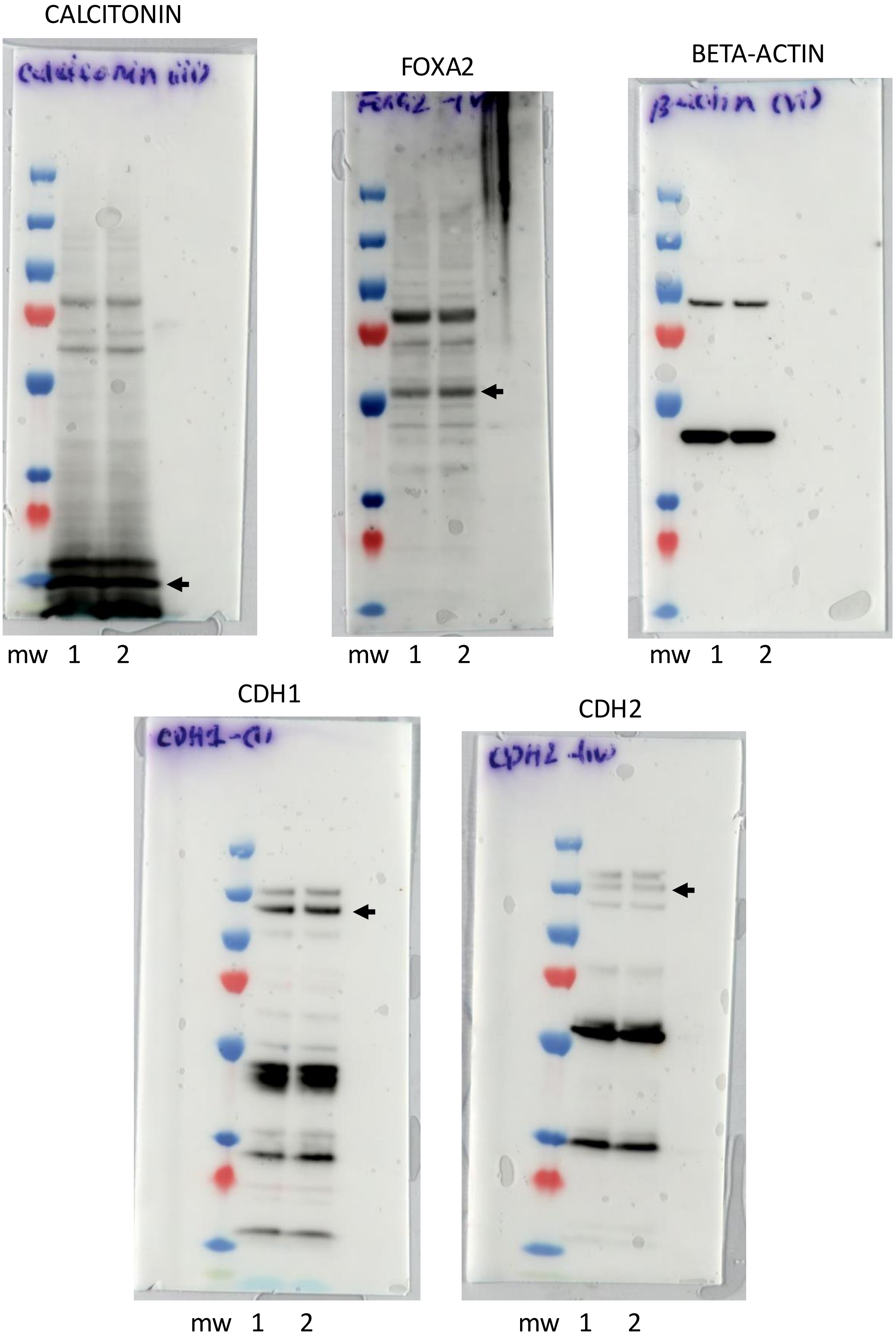

Uncropped Western blot data of Fig. 9c. Supplementary information to main Fig. 9c referring to marker gene expression in TT cells: Calcitonin, E-cadherin/Cdh1, N-cadherin/Cdh2, Foxa2 and beta-actin. Doublet samples analyzed. Molecular weight (mw) ladder in color is included in all blots. Arrows indicate bands with the expected molecular weight for each marker protein. Notably, blots of Cdh1 and Cdh2 reveal multiple bands consistent with phosphorylated cadherin variants, which display a higher molecular weight than the native protein, and some strongly labeled low molecular-weight peptides presumably reflecting high cadherin turnover.
