# Supplementary material for: Resolving thyroid lineage cell trajectories merging into a dual endocrine gland in mammals
Source: Nat Commun. 2026 May 25;17:6811. doi: 10.1038/s41467-026-73385-6 (PMC13385830; doi:10.1038/s41467-026-73385-6)
Supplement: Supplementary file 11 — Reporting Summary [file 41467_2026_73385_MOESM11_ESM.pdf]

nature portfolio

Corresponding author(s): Mikael Nilsson
Rene Maehr

Last updated by author(s): Mikael Nilsson (on April 9, 2026)

Reporting Summary

Nature Portfolio wishes to improve the reproducibility of the work that we publish. This form provides structure for consistency and transparency in reporting. For further information on Nature Portfolio policies, see our Editorial Policies and the Editorial Policy Checklist.

Statistics

For all statistical analyses, confirm that the following items are present in the figure legend, table legend, main text, or Methods section.

n/a	Confirmed	
[ ]	[x]	The exact sample size (*n*) for each experimental group/condition, given as a discrete number and unit of measurement
[ ]	[x]	A statement on whether measurements were taken from distinct samples or whether the same sample was measured repeatedly
[ ]	[x]	The statistical test(s) used AND whether they are one- or two-sided<br>*Only common tests should be described solely by name; describe more complex techniques in the Methods section.*
[ ]	[x]	A description of all covariates tested
[ ]	[x]	A description of any assumptions or corrections, such as tests of normality and adjustment for multiple comparisons
[ ]	[x]	A full description of the statistical parameters including central tendency (e.g. means) or other basic estimates (e.g. regression coefficient) AND variation (e.g. standard deviation) or associated estimates of uncertainty (e.g. confidence intervals)
[ ]	[x]	For null hypothesis testing, the test statistic (e.g. *F*, *t*, *r*) with confidence intervals, effect sizes, degrees of freedom and *P* value noted<br>*Give P values as exact values whenever suitable.*
[x]	[ ]	For Bayesian analysis, information on the choice of priors and Markov chain Monte Carlo settings
[x]	[ ]	For hierarchical and complex designs, identification of the appropriate level for tests and full reporting of outcomes
[ ]	[x]	Estimates of effect sizes (e.g. Cohen's *d*, Pearson's *r*), indicating how they were calculated

Our web collection on statistics for biologists contains articles on many of the points above.

Software and code

Policy information about availability of computer code

Data collection	No software was used.
Data analysis	We used the following software and databases for data analysis : Python (3.8.17, 3.12.9), scanpy (1.9.3, 1.11.1), anndata (0.9.1, 0.11.4), jupyter notebook (1.0.0), numpy (1.26.4), scipy (1.15.2), leiden (0.10.1), igraph (0.10.6), pandas (1.5.3, 2.0.3), seaborn (0.12.2, 0.13.2), matplotlib (3.10.1), celloracle (0.6.6), scvelo (0.3.3), CellRank (2.0.6), Palantir (1.4.1), harmonyTS (0.1.5) moscot (0.4.2), NIS-elements 6.0, Affinity Designer 2.

For manuscripts utilizing custom algorithms or software that are central to the research but not yet described in published literature, software must be made available to editors and reviewers. We strongly encourage code deposition in a community repository (e.g. GitHub). See the Nature Portfolio guidelines for submitting code & software for further information.

Data

Policy information about availability of data

All manuscripts must include a data availability statement. This statement should provide the following information, where applicable:

- Accession codes, unique identifiers, or web links for publicly available datasets
- A description of any restrictions on data availability
- For clinical datasets or third party data, please ensure that the statement adheres to our policy

This paper analyzes existing publicly available data in the Gene Expression Omnibus (GEO) database under accession codes “GSE182135” (scRNA atlas) and “GSE182134” (scATAC atlas).
We also used data analyzed in: https:/github.com/maehrlab/pharyngeal_endoderm_development.
Custome code is available at: https://github.com/maehrlab/ThyroidUBB_Lineage.

Research involving human participants, their data, or biological material

Policy information about studies with human participants or human data. See also policy information about sex, gender (identity/presentation), and sexual orientation and race, ethnicity and racism.

Reporting on sex and gender	n/a
Reporting on race, ethnicity, or other socially relevant groupings	n/a
Population characteristics	n/a
Recruitment	n/a
Ethics oversight	n/a

Note that full information on the approval of the study protocol must also be provided in the manuscript.

Field-specific reporting

Please select the one below that is the best fit for your research. If you are not sure, read the appropriate sections before making your selection.

☒ Life sciences ☐ Behavioural & social sciences ☐ Ecological, evolutionary & environmental sciences

For a reference copy of the document with all sections, see nature.com/documents/nr-reporting-summary-flat.pdf

Life sciences study design

All studies must disclose on these points even when the disclosure is negative.

Sample size	The study relied on previously published scRNAseq and scATACseq datasets. For these, we used the sample size provided via the published datasets which allowed the original authors to reach their conclusions. Validation experiments on mouse embryos and adults comprised 10 or more individuals for each type of experiment and age being analysed. Animal groups for comparison consisted of 3 or more individuals per experiment.
Data exclusions	For scRNAseq analyses of the UBB /thyroid lineages, we excluded clusters not belonging to those lineages based on the expression of marker genes and using clusters defined according to the original manuscript. Our rationale is restated in the methods. No data obtained from validation experiments were excluded.
Replication	All the computational findings are reproducible based on code available in the Github repository (see Code availability statement).<br>All data obtained by imaging (microscopy or western blotting) were repeated at least three time for each type of eperiment with similar results.
Randomization	All single cell analyses were performed in a randomized manner meaning cells from all samples were combined, analyzed and allocated into clusters at a resolution which captured different cell types based on differentially expressed markers. Experimental sample was controlled for to overcome batch effects for the analysis of the scRNA dataset. Animals for validation experiments were included in a randomized manner based on current breeding and available litters.
Blinding	For the scRNAseq datasets, investigators were not blinded to experimental groups after processing as this would preclude grouping of replicates for analysis.Morphological analysis e.g. of genotype-phenotype correlations was primarily blinded to investigators.Group allocation e.g. of mutant mice with different genotypes was not blinded to coordinator of experimental setup.

Reporting for specific materials, systems and methods

We require information from authors about some types of materials, experimental systems and methods used in many studies. Here, indicate whether each material, system or method listed is relevant to your study. If you are not sure if a list item applies to your research, read the appropriate section before selecting a response.

Materials & experimental systems

n/a	Involved in the study
☐	☒ Antibodies
☐	☒ Eukaryotic cell lines
☒	☐ Palaeontology and archaeology
☐	☒ Animals and other organisms
☐	☒ Clinical data
☒	☐ Dual use research of concern
☒	☐ Plants

Methods

n/a	Involved in the study
☒	☐ ChIP-seq
☒	☐ Flow cytometry
☒	☐ MRI-based neuroimaging

Antibodies

Antibodies used	rabbit anti-NKX2-1/TTF-1 (PA0100/1:1000; Biopat; ab227652/1:100; Abcam), rabbit anti-PAX8 (No 10336-1-AP/1:2000; Proteintech Europe), guinea pig anti-FOXA1 (kindly provided by Jeffrey Whitsett, Cincinnati Children's Hospital, OH, USA; 1:2000; applied for mouse tissue only), mouse anti-FOXA1 (No WMAB-2F83/1:1000; Seven Hills Bioreagents; applied for human specimens only), rabbit anti-FOXA2 (WRAB-FOXA2/1:2000; Seven Hills), rat anti-E-cadherin/CDH1 (13-1900/1:4000; Novex/Life Technologies), rabbit anti-N-cadherin/CDH2 (ab18203/1:500 for IHC, 1:200 for IF; Abcam), rabbit anti-laminin/LAM (L9393/1:500; Sigma-Aldrich), chicken anti-laminin/LAM (ab14055/1:500; Abcam), rabbit anti-COL4A1 (ab6586/1:400; Abcam), rat anti-CD31 (550274/1:250; Pharmingen), rabbit anti-thyroglobulin/TG (A0251/1:5000; Agilent), rabbit anti-calcitonin/CALC (102480/1:500; Agilent), rabbit anti-pericentrin/PCNT (ab4448/1:1000; Abcam), rabbit anti-Ki67 (ab15580/1:500; Abcam), and Armenian hamster anti-mucin1/MUC1 (CT2 monoclonal against aa 239-255 (SSLSYTNPAVAATSANL) of the cytoplasmic tail of MUC1, 1:1000; gift from Cathy Madsen at Sandra Gendler Lab, Mayo Clinic; also available at ThermoFisher MA5-11202); rabbit anti-HEYL (No 15679-AP/1:1000; ThermoFisher).<br>For antibody validation see the respective manufacturer´s websites.
Validation	Immunofluorescence, immunohistochemistry and western blot analysis were performed according to best laboratory practice and the indicated supplier´s instructions. Obtained results were validated by control immunostaining with omitted or irrelevant primary antibodies and evaluation of the expected expression patterns in other embryonic and adult tissues.

Eukaryotic cell lines

Policy information about cell lines and Sex and Gender in Research

Cell line source(s)	A human TT cell line established from a White 77 years old female with medullary thyroid carcinoma. Source: ATCC (Cat nr: CRL-1803). This cell line is not listed as being known misidentified.
Authentication	Expression analysis of known biomarkers (e.g. calcitonin) by Western blotting and immunofluorescence was performed along with the indicated validation experiment.
Mycoplasma contamination	The used cell line was not recently tested for mycoplasma contamination.
Commonly misidentified lines (See ICLAC register)	n/a

Animals and other research organisms

Policy information about studies involving animals; ARRIVE guidelines recommended for reporting animal research, and Sex and Gender in Research

Laboratory animals	We used wildtype C57BL/6J mice (obtained from Taconic Biosciences), heterozygous Nkx2-1CtreERT2 and TgCreET2;BrafCA mice (obtained from Jackson Laboratories), and Nkx2-1 and Pax8 knockout mice (obtained from the animal facility at University of Naples Federico II, Naples, Italy), all of the same strain background. Animals used for validation experiments were kept at the animal facility Laboratory of Experimental Medicine (EBM) at Sahlgrenska University. Housing conditions comprised regular temperature and humidity and dark/light cycle. Mouse embryos were sampled and analysed at embryonic days E9.5-E18.5. Adult mice were sampled and analysed at 6 weeks or older.
Wild animals	The study did not involve wild animals.
Reporting on sex	The study comprised mouse embryos of both sexes unselected and without further considerations. There are no indications of sex differences in embryonic development of the thyroid gland. The study comprised adult mice of both sexes unselected. We previously reported minor sex-biased differences in thyroid growth in response to mutant Braf, which were not considered in this study.
Field-collected samples	The study did not involve samples collected from the field.
Ethics oversight	Animal experiments were approved by: the regional ethical committee (Approval No 26-2013 and 5.8.18-03925/2018) according to European standards and national regulations provided by the Swedish Agriculture Agency; the local ethical committee "Comitato

	Etico per la Sperimentazione Animale" (CESA) in accordance with the regulations and guidelines of Italy and the European Union; the Institutional Animal Care and Use Committee (Approval protocol #2384) according to the regulatory standards defined by the National Institutes of Health and the University of Massachusetts.

Note that full information on the approval of the study protocol must also be provided in the manuscript.

Clinical data

Policy information about clinical studies

All manuscripts should comply with the ICMJE guidelines for publication of clinical research and a completed CONSORT checklist must be included with all submissions.

Clinical trial registration	n/a
Study protocol	Validation of gene expression in FFPE-samples of human thyroid cancer.
Data collection	One patient´s surgically excised thyroid tumor was selected for histopathological and immunohistochemical analyses. The patient previously participated in another study based on patient-derived xenografts for which informed consent was received (J Pathol, 266 (4-5): p. 481-494, 2025).
Outcomes	Obtained results validated present in silico findings in mice.

Plants

Seed stocks	n/a
Novel plant genotypes	n/a
Authentication	n/a
