# Supplementary material for: Resolving thyroid lineage cell trajectories merging into a dual endocrine gland in mammals
Source: Nat Commun. 2026 May 25;17:6811. doi: 10.1038/s41467-026-73385-6 (PMC13385830; doi:10.1038/s41467-026-73385-6)
Supplement: Supplementary file 12 — Transparent Peer Review file [file 41467_2026_73385_MOESM12_ESM.pdf]

nature portfolio

Peer Review File

Resolving thyroid lineage cell trajectories merging into a dual endocrine gland in mammals

Corresponding Author: Professor Mikael Nilsson

This file contains all reviewer reports in order by version, followed by all author rebuttals in order by version.

Version 0:

Reviewer comments:

Reviewer #2

(Remarks to the Author)
The manuscript by Lobo et al describes how UBB, the endoderm originated structure, acquires neuroendocrine features and merges with the thyroid, by using bioinformatics analysis of scRNAseq data obtained from anterior endoderm at early embryonic days (E9.5-E12.5). They carried out IHC to support the results obtained by these computationally analyzed results. The paper is well-written, and the results presented are interesting. This paper may finally be able to stop the widespread misconception of neuroendocrine origin of UBB. There are several points that need clarification as listed below.

In their writing, sometimes the use of ", (comma)" would help to give the readers a necessary break to better understand the contents due to immense amount of data presented and discussed.

Line 97: "see Fig. 5 for experiments using PAX8 as biomarker in this paper" is not clear since Fig. 5 does not have results with PAX8.

Fig. 4B: can you make the colors blue (G1), green (S) and orange (G2M) stronger? It would help to differentiate from gray.

All figures: include the number over the scale bar. If the scale bar is for 25um, include "25um" over the scale bar in the figure itself, not in the legend. This makes easier for the reader to understand the difference in sizes and perspective without having to go to legend all the time.

Line 473: It says "We further addressed the role of EMT in thyroid development by comparing single-cell expression of Cdh1 and Cdh2, which are important determinants of the EMT process typically antagonized by E-cadherin/Cdh1 and promoted by N-cadherin/Cdh2 [53, 54]."
Then, "It was not possible to visualize CDH2 by immunofluorescence before the Ubb merged with the thyroid presumably due to N-cadherin/Cdh2 protein expression is yet very low in the developing Ubb (data not shown). However, once thyroid lobes are established scattered Cdh2+ cells are readily observed alongside the branching thyroid epithelium similarly to cells expressing calcitonin (Figs. 5j and k)."
Since Cdh2 promotes EMT, should it not be expressed during the UBB before/during merging stage? Then, when thyroid lobes are stablished, Cdh2 are detected? If it is UBB that is moving down to reach thyroid, I'm confused. If it is thyroid precursor cells that are migrating to merge into UBB it makes sense. Please provide more detailed interpretation.

Line 493: It says "In the adult thyroid, CDH2+ cells attain a parafollicular position identical to that of resident C-cells (Supplementary Figs. 13a and b). Equal numbers of cells expressing these biomarkers indicate that N-cadherin/Cdh2 no longer identifies an immature cell stage but rather defines the neuroendocrine phenotype of thyroid C-cells in adult mice." Does CALCA(+) C-cell co-express N-cad? Probably it would be better to have a higher magnification image from Supplementary Figs. 13a and b showing that these proteins do NOT make part of follicles, or show IF results for NKX2-1 + CALCA and NKX2-1 and N-cad, or NKX2-1 + CALCA + N-CAD.

Sup. Fig. 17 legend: counterStaining of nuclei is missing the S in the spelling.

Line 663: It says "In Nkx2-1 null heterozygous mutants examined by serial sectioning at E15.5, the thyroid lobes are considerably smaller than in normal embryos of the same age (see Figs. 1e and f for comparison) and largely consists of

NKX2-1low cells surrounded by minor numbers of NKX2-1high cells (Supplementary Fig. 18a)."
They indicate to compare Fig. 1e and f with Supp. Fig 18a, but they do not present the same marker (in the case, NKN2-1 was indicated). Besides, scale bars are different in size, 25um on Supp. Fig. 18a and 50um on Fig. 1e and f, making the comparison difficult. They should have a control on Supp. Fig. 18 in the same scale that allows to easily compare the size. The way they try to show is not clear.

Line 664-665: se Figs. 1e and f for comparison. Fig. 1 does not show the results of Nkx2-1+/- mice.

Fig. 8 needs a control for panel d-k. They compare to Figure 4, but, again, do not analyze the same markers nor same Embryonic times.

Reviewer #3

(Remarks to the Author)
This interesting study by Lobo et al. effectively uses single cell RNA sequencing approach to distinguish thyroid and C-cell lineages, enabling high-resolution insights into gene expression patterns and lineage-specific trajectories. The manuscript highlights specific transcription factors and regulatory networks, shedding light on the molecular mechanisms guiding thyroid and ultimobranchial body development. This helps understand how gene regulatory cues direct lineage commitment and cellular differentiation. Through gene expression mapping at various embryonic stages, the study identifies lineage-specific changes over time, highlighting the dynamics of cellular differentiation and thyroid organ formation.
While I observe a highly detailed descriptive framework, there is weakness in this study, which is the use of experimentally unvalidated in silico knockout models interpreted as an additional layer of validation for identifying critical genes in the thyroid and Ubb lineage commitment. These models do not perform at the level of trust, and need be either additionally validated with orthogonal experimental approaches (at least some) or discussed with a warning. The performance of CellOracle is very far from 100%. Such predictions from in silico simulations shall be used only to narrow down the hypothesis and run focused experiments, which I expect from the authors during this revision. Overall, the experimental validations of identified regulatory interactions are limited, reducing the direct applicability of single cell transcriptomics-based findings. While the authors provide a lot of correlative gene expression data, numerous conclusions regarding gene function and regulatory network dynamics would benefit from more functional experiments attempted in ex vivo systems, such as explants of developing endodermal derivatives. Also, it would be very interesting to see the phenotypes of Pax8 and Nkx2-1 mutants at the single cell level, in addition to in silico ko simulations (I really doubt these results will be in coherence).

Reviewer #4

(Remarks to the Author)
Macrina et al. present a study that investigates the early development and colonization of two endocrine cell types in the thyroid using single-cell transcriptomics combined with morphological experiments. This work addresses an important fundamental question: how the thyroid primordium develops independently, which makes it a novel and valuable contribution. Additionally, by linking developmental biology findings to the pathogenesis of a specific subtype of thyroid carcinoma, the study also carries some clinical relevance.
Overall, this is a well-executed study with strong bioinformatics analyses supported by morphological validation. We suggested that the authors should describe the insignificant results of bioinformatics analysis that have not been preliminarily confirmed by experiments in the supplementary. However, several aspects require clarification and further validation to improve the rigor and clarity of the manuscript.
1. Trajectory Analysis of Single-Cell Data
The authors identified 5,904 cells for further analysis. To better reconstruct the developmental process at the single-cell level, I strongly recommend performing pseudotime trajectory analysis and RNA velocity analysis. These approaches would help infer cell lineage evolution and differentiation trajectories, strengthening the overall conclusions.
2. Batch Effect and Sample Correction
The UMAP clustering shows substantial differences in cell population proportions across time points. For instance, clusters 0, 5, and 7 are almost exclusively detected at E9.5, while cluster 9 is mainly present at E10.5.
It remains unclear whether this variation is biological (reflecting developmental heterogeneity) or technical (due to sample handling, processing time, or reagent batch effects).
The authors should clarify which methods were used for batch correction in the single-cell RNA sequencing analysis. Was Harmony applied?
I suggest comparing at least three different batch correction methods (e.g., Harmony, MNN, and Seurat integration) to ensure the robustness of the clustering and lineage inference.
3. Expression Representation in Figure 1q
The claim that Nkx2-1, Foxa2, Meox1, Ripply3, Ascl1, and Calca are co-expressed in clusters 8, 6, and 4 appears inaccurate.
Foxa2, Ascl1, and Calca are primarily enriched in cluster 4 rather than across all three clusters.
Please clarify this representation to ensure accuracy.
4. Inconsistent Interpretation of Ripply3 Expression
The manuscript states that "Ripply3 predominates early and gradually diminishes." However, Figure 1r suggests that Ripply3 expression peaks at E10.5 before decreasing.
This discrepancy needs to be corrected.

5. Disappearance of Cluster 0 at E12.5
Figure 1r suggests that Cluster 0 disappears entirely by E12.5.
The authors should confirm whether Cluster 0 is completely absent at this stage and discuss its biological significance.
6. Clarification of Numerical Values in Lines 154-157
The meaning of the numbers in the following sentence is unclear:
"Interestingly, upregulation of Ripply3 restricted to clusters 7 (x 36.6), 8 (x 15.1), and 9 (x 7.1) mimics that of Hoxb1 (cluster 7: 33.9; cluster 8: x 18.4; cluster 9: 7.6), which is specifically expressed in the last most inferior pharyngeal pouch."
Do these numbers represent fold changes, normalized expression values, or statistical measures?
This needs to be explicitly clarified.
7. Interpretation of UMAP Data in Supplementary Figure 2
Supplementary Figure 2 presents UMAP plots showing gene expression at different time points, but the meaning of the gray dots is unclear.
If these dots represent missing data points, I suggest removing them to make the plots clearer.
8. Misleading Conclusion in Lines 154-157
The statement "Cluster 1 likely represents a pool of undifferentiated progenitor cells committed to a thyroid fate" is based on Figure 2c.
However, pathway analysis suggests Cluster 3 is more closely related to Cluster 2, while Cluster 1 is more distant.
I recommend using Figure 3a rather than Figure 2c to support this conclusion.
9. Visualization Improvement for Figure 3c
Figure 3c is difficult to interpret.
Consider revising the format to make the data more intuitive.
10. UMAP Visualization of Ki67 Expression in Figure 4a
The manuscript discusses Ki67 index, but a corresponding UMAP visualization is missing.
Please include a UMAP plot of Ki67 expression.
11. Logical Flow of the Results Section
The results section lacks clear organization and jumps between different topics.
For example, the text first discusses thyroid clusters, then abruptly shifts to Ubb clusters (Figures 4g and 4k).
Similarly, the immunofluorescence results are not structured systematically.
I suggest reorganizing this section for better logical flow.
12. Contradiction in Foxa1/Foxa2 Expression
The manuscript states:
"The large proportion of cells with undetectable or low Foxa1/Foxa2 expression likely reflects permanent repression in thyroid lineage cells and transient downregulation in the fourth pharyngeal pouch."
However, Figure 4o shows that Foxa1/Foxa2 are still expressed in thyroid clusters at E12.5, sometimes even higher than at E9.5.
This inconsistency should be resolved.
13. Experimental Validation of Foxa2's Role in Ubb Development
While bioinformatics analysis suggests Foxa2 regulates Ubb differentiation, this needs experimental validation.
I strongly recommend functional in vivo or in vitro experiments to support this claim.
14. Relevance of "Ubb Cells of Different Maturity" Analysis
The bioinformatics analysis in this section appears weakly connected to the study's main focus.
Consider removing or condensing this part.
15. CDH1 and C-cell Localization Validation
Figure 5 immunofluorescence and Supplementary Figure 13 immunohistochemistry suggest that CDH1-expressing cells may be C-cells.
This should be confirmed using dual-color immunofluorescence or single-cell co-expression analysis of CDH2 and C-cell markers.
16. Validation of ECM Differences Between Thyroid and Ubb Lineages
Figure 6 suggests distinct ECM compositions, but this is based solely on bioinformatics data.
Morphological validation (e.g., immunofluorescence for ECM proteins) should be provided.
17. Manuscript Structure and Focus
The manuscript covers too many topics, ranging from thyroid and C-cell development to thyroid cancer pathology.
Some sections lack experimental validation, and the number of figures is excessive.
I suggest streamlining the manuscript, focusing on a clear central hypothesis, and ensuring all key claims are well-supported by experimental data.
Additionally, some figures appear in a disordered manner, making it difficult to follow the results logically.
The manuscript would benefit from restructuring for clarity and coherence.

Version 1:

Reviewer comments:

Reviewer #2

(Remarks to the Author)
The revised manuscript by Lobo, et al now contains multiple experimental results using RNA scope, immunofluorescence

and western blotting, which clearly validate computational analysis results. The manuscript is now ready to be accepted.

Reviewer #3

(Remarks to the Author)
I do not believe the authors have sufficiently addressed my comments regarding the need to expand the analysis of mutants and to provide additional functional validations. These points remain important, and I believe the authors could address them in a further round of peer review. While I find the current response largely unsatisfactory, I am not inclined to recommend rejection at this stage.

Reviewer #4

(Remarks to the Author)
The revision addresses my earlier concerns and substantially strengthens the manuscript. The additions include (i) new pseudotime/trajectory analyses (Palantir and moscot implemented within CellRank2), with an explanation of the limited performance of scVelo and CytoTRACE on these data; (ii) added RNAscope, immunofluorescence and Western blot validations at key embryonic stages; and (iii) a clearer, developmentally ordered presentation of Results with an extensive figure/legend re-organization. These changes improve the linkage between single-cell inferences and in vivo morphogenesis.
Minor recommendation for the Discussion.
Please add a brief paragraph situating your EMT-related observations within recent literature on epithelial plasticity in thyroid development, e.g., PMID 38057310. That study reports an NF-κB–activated thyrocyte subpopulation with partial mesenchymal features and shows that myeloid-derived TNF-α promotes thyrocyte migration and folliculogenesis in zebrafish/mouse. A short comparison would help readers: (i) migrating thyrocytes in postnatal/embryonic contexts vs. UBB/C-cell precursors in your embryonic fusion model; (ii) upstream cues (TNF-α–NF-κB axis in thyrocytes) vs. the GRNs and basement-membrane remodeling you define; and (iii) whether the states you observe align best with EMT, collective migration, or a hybrid epithelial/mesenchymal program (your response already discusses collective migration vs. EMT timing). Two to three sentences would suffice; no new experiments are needed.
With the above brief addition to the Discussion and routine checks, I support acceptance.

Version 2:

Reviewer comments:

Reviewer #3

(Remarks to the Author)
The authors sufficiently addressed my concerns. The manuscript can be accepted for publication.

Open Access This Peer Review File is licensed under a Creative Commons Attribution 4.0 International License, which permits use, sharing, adaptation, distribution and reproduction in any medium or format, as long as you give appropriate credit to the original author(s) and the source, provide a link to the Creative Commons license, and indicate if changes were

made.
In cases where reviewers are anonymous, credit should be given to 'Anonymous Referee' and the source.
The images or other third party material in this Peer Review File are included in the article’s Creative Commons license, unless indicated otherwise in a credit line to the material. If material is not included in the article’s Creative Commons license and your intended use is not permitted by statutory regulation or exceeds the permitted use, you will need to obtain permission directly from the copyright holder.
To view a copy of this license, visit https://creativecommons.org/licenses/by/4.0/

We thank all reviewers for valuable comments and suggestions of improvement.

To address the reviewers' comments, the revised manuscript contains additional supporting data both computational, mainly pseudotime and trajectory interference analysis, and experimental, including multiple validation experiments comprising RNAscope, immunofluorescence and Western blot analysis.

The order of presentation of Results is rearranged to accommodate the new data and for improved clarity, adhering better to the natural time course of thyroid development than in the previous version. For this reason, both main and supplementary figures to variable extent contain new data and/or swapped data. Some previous data, surplus or of less importance, have been deleted.

For your guidance and overview, revised data sets comprise:

Main figures 1-9

- Fig. 1.
  New: Fig. 1b-f.
  Swapped: Fig. 1l, m, n, p (from previous fig. 2).
  To Supplementary: previous fig. 1b.
  Deleted: previous fig. 1b-k (replaced by
  RNAscope images), fig. q (part of) and fig. 1r
- Fig. 2. New: Fig. 2a-f, i-l
- Figs. 3 and 4 are split from previous fig. 3.
  New: Figs. 3g-i and 4f-n.
  Swapped: Fig. 3c (from previous Supplementary Fig. 5), figs. 3f and 4d (from previous Supplementary Fig.6).
  Deleted: previous fig. 1c
- Fig. 5 are merged from previous figs. 6 and 7.
  New: Fig. 5c-m.
  Swapped: Fig. 5a, b (from previous fig. 6) and fig. 5n-r (from previous fig. 7).
  Deleted: previous fig. 6a, d, e (part of)
- Fig. 6 is a combo of previous fig. 7 and 8.
  New: Fig. 6b, h, i-l (part of), n and o, p (top images new, the others rearranged).
  Swapped: Fig. 6b-c (from previous Supplementary Fig. 17).
  To Supplementary: previous fig. 8a (to new Supplementary Fig. 12).
  Deleted: previous fig. 6a-e (replaced by Supplementary Fig. 11) and fig.6o, p (replaced by new Fig. 8d, e).
- Fig. 7 adopted from previous fig. 4.
  New: Fig. 7a-d, g (part of), h, p-u.
  Swapped: Fig. 7e, f (from previous Supplementary Fig. 9a, b in which (e) is truncated due to space limitation) and j (from Previous Supplementary Fig. 8b).
  To Supplementary: previous fig. 4a-c and fig. 4o (both to new Supplementary Fig. 6 and modified).
  Deleted: previous fig. 4g-i, k (replaced by new images in new Supplementary Fig.

6), fig. 4l and n

- Fig. 8 adopted from previous fig. 5.
  New: Fig. 8a-f, m-q. Swapped: -.
  To Supplementary: previous fig. 5a-c (to new Supplementary Fig. 7).
  Deleted: previous fig. 5g (part of), 5j-l (replaced by better image)
- Fig. 9.
  New: Fig. 9b, c, f, h, i, m-o, q (replacing previous similar images).
  Swapped: Fig. 9a (from previous Supplementary Fig. 13).
  Deleted: previous fig. 9e-f and fig. 9g, h, k (replaced by better images)

Supplementary Figures (S): total number reduced from 18 to 15

- New: S6bc, S8, S10 (modified from former Figs. 7a-e and S17), S11 (modified from former Fig. 7a), and S13
- Identical: S1, S2 (former Fig. S3), S3 (former Fig. S2), and S14 (former Fig. S9)
- Modified: S4 (former fig. S14 modified), S5 (combo of former figs. 4a-c and 4o modified), S6 (combo of former Fig. S11 and Fig. 5 modified), S7 (former Fig. S15 modified), S9 (former Fig. 16 modified), S12 (former Fig. S18 modified) and S15 (former Fig. S12 modified)
- Deleted: S4, S8a, S10c-e, S13b, s15b, S17c-e, S18b
- Swapped to Main: former Fig. S5, S6, S7, S8b, S10ab, S13a

Supplementary Datasets 1-3 are new
SD 1: Rank list of thyroid lineage drivers
SD 2: Rank list of Ubb/C-cell lineage drivers
SD 3: Uncropped Western blots

Response to Reviewer #2

The manuscript by Lobo et al describes how UBB, the endoderm originated structure, acquires neuroendocrine features and merges with the thyroid, by using bioinformatics analysis of scRNAseq data obtained from anterior endoderm at early embryonic days (E9.5-E12.5). They carried out IHC to support the results obtained by these computationally analyzed results. The paper is well-written, and the results presented are interesting. This paper may finally be able to stop the widespread misconception of neuroendocrine origin of UBB. There are several points that need clarification as listed below.

The authors thank the reviewer for appreciating our major finding of Ubb/C-cell lineage derivation from pharyngeal endoderm (and not neural crest as assumed before).

In their writing, sometimes the use of ", (comma)" would help to give the readers a necessary break to better understand the contents due to immense amount of data presented and discussed.

The text has been extensively revised for improved clarity and a more logical order of presentation.

Line 97: "see Fig. 5 for experiments using PAX8 as biomarker in this paper" is not clear since Fig. 5 does not have results with PAX8.

Thank you for noticing this error. In the revised manuscript new Pax8 data, based on RNAscope, pseudotime/trajectory inference and immunofluorescence, are provided in revised Fig. 1d, Fig. 2e, f, i-l, and Fig. 6i-f with explaining text in Results (lines 101-102; 133-137; 168-172; 176-192; 341-344) and corresponding figure legends.

Fig. 4B: can you make the colors blue (G1), green (S) and orange (G2M) stronger? It would help to differentiate from gray.

We now remade the plots with darker shades and without cell outlines for improved resolution, see revised Supplementary Fig. 5d.

All figures: include the number over the scale bar. If the scale bar is for 25um, include "25um" over the scale bar in the figure itself, not in the legend. This makes easier for the reader to understand the difference in sizes and perspective without having to go to legend all the time.

We have revised all figures to conform with a single or fewer variants of magnification (bar size). Adding numeric values as suggested would not conform with the journal´s format and we hope current modifications are sufficient to guide readers.

Line 473: It says “We further addressed the role of EMT in thyroid development by comparing single-cell expression of Cdh1 and Cdh2, which are important determinants of the EMT process typically antagonized by E-cadherin/Cdh1 and promoted by N-cadherin/Cdh2 [53, 54].”
Then, “It was not possible to visualize CDH2 by immunofluorescence before the Ubb merged with the thyroid presumably due to N-cadherin/Cdh2 protein expression is yet very low in the developing Ubb (data not shown). However, once thyroid lobes are established scattered Cdh2+ cells are readily observed alongside the branching thyroid epithelium similarly to cells expressing calcitonin (Figs. 5j and k).”
Since Cdh2 promotes EMT, should it not be expressed during the UBB before/during merging stage? Then, when thyroid lobes are stablished, Cdh2 are detected? If it is UBB that is moving down to reach thyroid, I'm confused. If it is thyroid precursor cells that are migrating to merge into UBB it makes sense. Please provide more detailed interpretation.

Based on our revision work, we provide new results using RNAscope (Fig. 8m, n) and additional immunofluorescence staining of Cdh2 at critical time points (Fig. 8o, p), which clearly show a dissociation of transcript and protein expression. Altogether, this indicates that onset of Cdh2 expression is not translated to protein until Ubb/C-cell lineage cells are able to migrate into the embryonic thyroid. Moreover, a more careful analysis of Cdh2+ cells reveals a striking heterogeneity (Fig. 8q, r-r´´) which, importantly, conform with scRNAseq data distinguishing C-cell precursors (Fig. 8i-k). From previous studies, we know that intrathyroidal migration of C-cells are commenced by heterotypic cell-cell interactions in part mediated by EphA4 (DOI: 10.1210/en.2010-0232). Present findings suggest that Cdh2 likely participates in this process.

Gross movement of embryonic tissues is not specifically addressed in the present study. Descent and lateral extension of the thyroid primordium is associated with the aortic sac and pharyngeal arch arteries, and disruption of vasculogenesis e.g. in Shh and Tbx1 mutant mice leads to ectopic thyroid localization. Whether the Ubbs actually migrate after being dissociated from pharyngeal endoderm or merely move passively due to morphogenesis of surrounding neck tissues is still an open question. If movement of primordia is an active process, it likely occurs by collective migration (mentioned in the manuscript; lines 82-83), which typically is not EMT-dependent. Moreover, as thoroughly discussed in the paper, our present findings suggest that although EMT is triggered in Ubb cells at an earlier stage it is not manufactured until contact with the thyroid primordium is established.

Line 493: It says “In the adult thyroid, CDH2+ cells attain a parafollicular position identical to that of resident C-cells (Supplementary Figs. 13a and b). Equal numbers of cells expressing these biomarkers indicate that N-cadherin/Cdh2 no longer identifies an immature cell stage but rather defines the neuroendocrine phenotype of thyroid C-cells in adult mice.”
Does CALCA(+) C-cell co-express N-cad? Probably it would be better to have a higher magnification image from Supplementary Figs. 13a and b showing that these proteins do

NOT make part of follicles, or show IF results for NKX2-1 + CALCA and NKX2-1 and N-cad, or NKX2-1 + CALCA + N-CAD.

Regarding the likelihood of calcitonin and Cdh2 co-expression, it is not possible to investigate this by double immunofluorescence with available antibodies. However, we addressed this issue in TT cells, a cell line derived from a human medullary thyroid carcinoma that has been instrumental in many previous studies including by us (Johansson et al Development 2015). In the revised manuscript, we provide western blot data that unequivocally show expression of CALCITONIN, CDH1, CDH2, FOXA1 AND FOXA2 (Fig. 9c; Supplementary Dataset 3). The rationale for investigating this was primarily in comparison to accompanying data obtained from the CALCA/FOXA2 negative neuroendocrine tumor. However, since TT-cells are CALCA+/FOXA2+ and therefore maintain a differentiated state, we consider these findings together with revised Fig. 8 sufficient to conclude that Cdh2 is a C-cell lineage marker. As suggested, we also provide a high-power image of CDH2 IHC in adult mouse thyroid (revised Fig. 9b), which show that most CDH2+ cells are parafollicular whereas some are intraepithelial i.e. identical to the normal distribution of C-cells. The text has been revised accordingly (lines 482-485).

Sup. Fig. 17 legend: counterStaining of nuclei is missing the S in the spelling.

Supplementary Fig. 17 is deleted, whereas some images transferred to revised Fig. 8. Any spelling errors in remaining figure legends are now corrected.

Line 663: It says "In Nkx2-1 null heterozygous mutants examined by serial sectioning at E15.5, the thyroid lobes are considerably smaller than in normal embryos of the same age (see Figs. 1e and f for comparison) and largely consists of NKX2-1low cells surrounded by minor numbers of NKX2-1high cells (Supplementary Fig. 18a)."
They indicate to compare Fig. 1e and f with Supp. Fig 18a, but they do not present the same marker (in the case, NKN2-1 was indicated).

Thanks for this remark. In the revised manuscript, a better comparison of Nkx2-1+/- mutant and wildtype mice regarding thyroid (lobe) size is provided in new Fig. 6 (a combo of former Fig. 7 and 8), which shows NKX2-1 at E13.5 in wt (Fig. 6c) and mutant at E15.5 (Fig. 6p). Former Supplementary Fig. 18a with the same marker is now shown as Supplementary Fig. 12. Notably, NKX2-1 expression in wt at E15.5 is presented in new Fig. 8a. Since there are ample documentation of thyroid size difference between E13.5 (time of fusion with the Ubb) and E15.5 (branching morphogenesis of thyroid lineage) throughout the manuscript results, and a previous publication on the same topic is cited (Liang et al Development 2018), we think this information is sufficient to appreciate the reduced thyroid lobe size in Nkx2-1 null heterozygous mice.

Besides, scale bars are different in size, 25um on Supp. Fig. 18a and 50um on Fig. 1e and f, making the comparison difficult. They should have a control on Supp. Fig. 18 in the same scale that allows to easily compare the size. The way they try to show is not clear.

Scale bars are conformed to 25 µm in revised Fig. 6 and Supplementary Fig. 12 to support comparison. With reference to Fig. 8a in the previous comment above, since it is low magnification and primarily intended to compare with the adjoining Fig. 8b, d and e, we think 100 µm bar size is more appropriate here.

Line 664-665: se Figs. 1e and f for comparison. Fig. 1 does not show the results of Nkx2-1+/- mice.

See comments and explanation above. Overall, the presentation and interpretation of data obtained from Nkx2-1$^{+/-}$ mutant mice are revised (lines 358-373 of revised manuscript and Fig. 6).

Fig. 8 needs a control for panel d-k. They compare to Figure 4, but, again, do not analyze the same markers nor same Embryonic times.

We thank the reviewer for pointing out this inconsistency. In the revised manuscript we explain why E15.5 was chosen for mutant analysis (lines 349-352). A suitable age-matched control group does not exist since at this stage of development the Ubb is already merged with and partially dissolved within the embryonic thyroid. As argued above, we agreed to that comparison with Ubb parameters at E13.0 is not optimal but nonetheless informative. We would like to emphasize, as more thoroughly shown in revised Fig. 6, the most important findings on laminin dynamics are evident by comparing the two age-matched mutants.

Response to Reviewer #3

This interesting study by Lobo et al. effectively uses single cell RNA sequencing approach to distinguish thyroid and C-cell lineages, enabling high-resolution insights into gene expression patterns and lineage-specific trajectories. The manuscript highlights specific transcription factors and regulatory networks, shedding light on the molecular mechanisms guiding thyroid and ultimobranchial body development. This helps understand how gene regulatory cues direct lineage commitment and cellular differentiation. Through gene expression mapping at various embryonic stages, the study identifies lineage-specific changes over time, highlighting the dynamics of cellular differentiation and thyroid organ formation.
While I observe a highly detailed descriptive framework, there is weakness in this study, which is the use of experimentally unvalidated in silico knockout models interpreted as an additional layer of validation for identifying critical genes in the thyroid and Ubb lineage commitment. These models do not perform at the level of trust, and need be either additionally validated with orthogonal experimental approaches (at least some) or discussed with a warning. The performance of CellOracle is very far from 100%. Such predictions from in silico simulations shall be used only to narrow down the hypothesis and run focused experiments, which I expect from the authors during this revision. Overall, the experimental validations of identified regulatory interactions are limited, reducing the direct applicability of single cell transcriptomics-based findings. While the authors provide a lot of correlative gene expression data, numerous conclusions regarding gene function and regulatory network dynamics would benefit from more functional experiments attempted in ex vivo systems, such as explants of developing endodermal derivatives. Also, it would be very interesting to see the phenotypes of and Nkx2-1 mutants at the single cell level, in addition to in silico ko simulations (I really doubt these results will be in coherence).

Thank you for the comprehensive and overall positive summary of findings.

We agree with the current limitations of *in silico* perturbation prediction approaches, such as CellOracle. We have addressed the current limitation in the Discussion (lines 547-550). In addition, as detailed below responding to similar concerns of reviewer #4, we conducted pseudotime and trajectory inference analysis (revised Fig. 2), which confirm and extend findings predicted from CellOracle.

We agree to the importance of validation experiments. To this end, we have conducted a series of in situ hybridizations by RNAscope at some critical time points (E12.0, E13.0, E15.5) to map the expression pattern of lineage-specific key and candidate genes. You find RNAscope images in support current scRNAseq data in both revised main figures (Nxk2-1/Pax8/Foxa2/Calca in Fig. 1, Nkx2-1/Heyl in Fig. 3, Prdm1 in Fig. 4, Col1a2/Col4a2 in Fig. 5, Foxa1/Foxa2 in Fig. 7 and Cdh2 in Fig. 8) and revised Supplementary figures (Col1a2/Col4a2 in Fig. S8 and Foxa1/Foxa2/Cdh2 in Fig. S13) and corresponding text changes. Validation by RNAscope and extended immunofluorescence staining of PAX8 (Fig. 2), collagen type I and IV (Fig. 5) and N-cadherin/CDH2 (Fig. 8) strongly support that thyroid and Ubb/C-cell lineage trajectories as characterized *in silico* correspond well to the different

developmental stages of thyroid organogenesis and therefore reliable. Altogether, these additions fill out some missing pieces that allow us to tie everything much better than in the first manuscript version.

As discussed in the revised manuscript (lines 547-553), the predicted GRNs and multitude of associated candidate target genes should be regarded as the first comprehensive framework of thyroid development that consider time course at single cell level, which likely will stimulate future in-depth studies. In the present work, we uncover lineage similarities and differences not previously recognized and demonstrate the usefulness of computed data in elaborating and revealing some new fundamental aspects of morphogenesis.

For *in vivo* perturbation experiments it will take >1 year to breed, develop and analyze conditional knockouts e.g. of Prdm1 from available transgenic mouse lines (Jackson). RNA interference should be possible with cell lines but does not recapitulate embryonic development and would be of limited value to the present work. Embryonic tissue explants have been found working models of mouse thyroid differentiation (DOI: 10.1016/j.mce.2020.110834 ; DOI: 10.3791/51641). However, although of certain validation value for functional experiments of the developmental process taking place after fusion of primordia, these models do not recapitulate earlier developmental stages and are therefore not directly comparable for validation of present scRNAseq data spanning from E9.5 to E12.5. Moreover, no one has so far successfully explanted the thyroid bud or Ubb presumably due to technical difficulties. If at all feasible, we foresee such efforts are not realistic to get started and manage within the time schedule allowed for manuscript revision. Notably, studies on embryonic thyroid explants are yet very few and cannot be considered a standard technology.

We agree that in depth analysis at single cell level of thyroid/Ubb in Nkx2-1 and Pax8 mutant mice would be of interest to more precisely characterize the role of these TFs in thyroid and Ubb development yet to be uncovered. However, we believe this is out of scope of the present work and more suitable for a separate and most likely long-standing project. In the present study, we examined these mutants with focus on post-fusion events in particular basement membrane dynamics, which on the basis of scRNAseq data were predicted to take place in wildtype mice. By imaging and morphometric quantification, we show that of Ubb laminin/basement membrane relies on a cell-autonomous mechanism that likely involves Nkx2-1. Independent of this, we tested the reliability of the computed thyroid and Ubb trajectories by Pax8 and Nkx2-1 knockout simulations. The predicted cluster streams of simulated Pax8ko vs Nkx2-1ko cells fit well with the differential in vivo expression pattern, and we think this notion is of sufficient value to keep experiments and findings as such, which is further clarified in the revised manuscript (lines 183-192). Thanks to the Pax8KO simulation, during revision work we were able to identify a second pool of Pax8+ cells present in the inferior pharyngeal pouch and the developing Ubb (revised Fig. 2j-l).

Altogether, we believe that the present in vivo data confirms important in silico results and thus demonstrates the usefulness of such a combined approach. To summarize, we successfully validated expression of candidate and previously known developmental genes by RNAscope at strategic embryonic time points (E12.0, E13.0) with reference to the time frame

of obtained scRNAseq results and organogenesis taking place after the thyroid primordium fused with Ubb (E15.5). By firmly establishing lineage-specific expression patterns in vivo (revised Fig. 1b-f, Fig. 3g-i, Fig. 4f-n, Fig. 5c-f, Fig. 7a, b, p-u and Fig. 8m, n) we are able to link with reasonable precision our *in silico* observations to morphogenesis and elucidate with some mechanistic insight how the mammalian thyroid becomes a composite organ consisting of two endocrine cell types of different embryonic origin, which is also reflected it the main title of the study, In the revised manuscript, we also extend our single cell lineage analysis by pseudotime and trajectory inference which arguably identifies thyroid and Ubb cell fate probabilities and terminal differentiation traits (for further details on this, please see response to Reviewer #4 below). In the revision work, we also generated comprehensive lists of lineage drivers (Supplementary Datasets 1 and 2), which likely will become a valuable resource to thyroid scientists in various fields of currently intense research (development, regenerative medicine, tumor biology).

Response to Reviewer #4

Macrina et al. present a study that investigates the early development and colonization of two endocrine cell types in the thyroid using single-cell transcriptomics combined with morphological experiments. This work addresses an important fundamental question: how the thyroid primordium develops independently, which makes it a novel and valuable contribution. Additionally, by linking developmental biology findings to the pathogenesis of a specific subtype of thyroid carcinoma, the study also carries some clinical relevance. Overall, this is a well-executed study with strong bioinformatics analyses supported by morphological validation. We suggested that the authors should describe the insignificant results of bioinformatics analysis that have not been preliminarily confirmed by experiments in the supplementary. However, several aspects require clarification and further validation to improve the rigor and clarity of the manuscript.

Thank you for appreciating the rationale of investigation and novelty of present findings.

As indicated above, the manuscript has been extensively revised which includes a better balance between main and supplementary data, as suggested. Some previous computed findings of central importance are now also validated by RNAscope and additional IF imaging.

Response to your specific points:

1. Trajectory analysis of single-cell data:

The authors identified 5,904 cells for further analysis. To better reconstruct the developmental process at the single-cell level, I strongly recommend performing pseudotime trajectory analysis and RNA velocity analysis. These approaches would help infer cell lineage evolution and differentiation trajectories, strengthening the overall conclusions.

We thank the reviewer for this suggestion and have performed detailed trajectory analyses. The tool Harmony from Nowotschin et al (PMID: 30959515; cited in revised manuscript) was used to join adjacent time points and visualize trajectories on a force directed embedding. To ensure the robustness of our findings we inferred trajectories using four methods as described below. Importantly, this analysis led to confirmatory identification of terminal states in addition to the identification of cells primed toward the thyroid and UBB lineages (Fig.2a-d), varying expression patterns of key markers along the lineage trajectories (Fig. 2e, f) and the unsupervised identification of known and novel lineage drivers (Supplementary dataset 1-2). Some of these findings regarding Pax8 are supported by previous *in silico* perturbation experiments and experimentally confirmed in intact mouse embryonic tissues. These exciting results are now presented in new Fig. 2 and corresponding text in the Results (lines 155-192).

Two of the methods Palantir and Moscot yielded consistent findings which have been included in the updated manuscript, as indicated above. We explain our analyses and results and discuss inadequacies of the other two methods here:

(a) Pseudotime analysis with Palantir (PMID: 30899105; cited): Utilizing cell connectivities from timepoints stitched together by Harmony, we run Palantir which models the differentiation as a stochastic process from a selected root cell to the explicitly defined Thyroid and UBB terminal clusters. We infer pseudotime and branch probabilities. We smooth gene expression along the lineages using MAGIC imputation (PMID: 29961576; cited) and visualize the resulting gene expression of key markers with heatmaps and UMAPs. Key lineage drivers are shown in Supplementary dataset 1 and 2 for thyroid and Ubb, respectively.
(b) Velocity analysis with scVelo (PMID: 32747759; cited): We infer spliced and unspliced counts and predict cell trajectories using the ratio of spliced and unspliced counts of a gene in a cell to represent the rate of expression change for that gene. We utilize scvelo's dynamic model to infer velocity. As described in Weiler et al (PMID: 36495456), scvelo is sometimes inaccurate, particularly for developmental data and combining cell-cell connectivity might improve performance. This was true in our dataset as well. However, even after combining the velocity kernel with a connectivity kernel, we did not achieve good results as we were unable to automatically predict terminal cell states and the direction of velocity arrows was not consistent with embryonic development (see Review Fig. 1 appendixed this document).
(c) Next, we apply Cytotrace (PMID: 31974247) which uses development potential measured by transcriptional diversity to align cells along pseudotime. We repeated downstream analyses as described in (a). This method is particularly useful for earlier embryonic timepoints (PMID: 31974247) which could explain the poor performance here as we observed the direction of inferred arrows was inconsistent with embryonic development (Review Fig. 2 appendixed this document).
(d) Finally, we applied an Optimal Transport based method moscot (PMID: 39843746; cited) which improves on the earlier widely used Waddington-OT (PMID: 30712874; cited). Moscot uses optimal transport to maps cells across timepoints and uses molecular similarity to map cells within timepoints while adjusting for cellular growth and death. Moscot correctly identifies the terminal states (UBB cluster 4 and Thyroid cluster 3). We infer lineage drivers and perform analyses as described in (a) and shown in revised Fig. 2d.

These analyses were implemented from within the CellRank 2 (PMID: 38871986; cited) framework.

2. Batch effect and sample correction:

The UMAP clustering shows substantial differences in cell population proportions across time points. For instance, clusters 0, 5, and 7 are almost exclusively detected at E9.5, while cluster 9 is mainly present at E10.5.
It remains unclear whether this variation is biological (reflecting developmental heterogeneity) or technical (due to sample handling, processing time, or reagent batch effects).
The authors should clarify which methods were used for batch correction in the single-cell RNA sequencing analysis. Was Harmony applied?
I suggest comparing at least three different batch correction methods (e.g., Harmony, MNN, and Seurat integration) to ensure the robustness of the clustering and lineage inference.

We thank the reviewer for the feedback. As noted in the Methods Section Single-cell RNAseq data analysis, (lines 698-728) we performed normalization, batch correction as well as correction for nuisance effects prior to UMAP embedding generation and leiden clustering. Normalization was performed with scran and batch correction was performed with combat using scanpy.pp.combat on the samples. Additionally, nuisance variables were corrected for, namely total counts, mitochondrial percentage, and phase, using scanpy regress_out.

Further, on the advice of the reviewer, to confirm that technical effects are not driving the clustering and downstream inference, we performed batch correction / data integration utilizing 4 state-of-the-art methods, namely Harmony (PMID: 31740819), BBKNN (PMID: 31400197), MNN (PMID: 29608177), and scVI (PMID: 30504886). We recomputed the nearest neighbor graph and UMAP embedding for each of the integration methods. We visualized the samples, embryonic day and clusters on each of the embeddings generated.

The goal of normalization and correction for this problem is to ensure that replicates at the same timepoint are well integrated while preserving biological effects across timepoints. We believe the current normalization-batch correction approach works well since replicates at the same timepoint are well integrated while developmental effects across embryonic day are preserved. We also believe that the other methods explored are less optimal. From the embeddings it appears that Harmony slightly overcorrects (high mixing across eday) and scVI seriously overcorrects (excessive mixing across Eday) while MNN undercorrects (replicates at the same timepoint are separated).

We do not believe that this analysis adds additional information to our main findings, so we only include it here (Review Fig. 3 appendixed this document).

3.Expression representation to Fig. 1q:

The claim that Nkx2-1, Foxa2, Meox1, Ripply3, Ascl1, and Calca are co-expressed in clusters 8, 6, and 4 appears inaccurate.
Foxa2, Ascl1, and Calca are primarily enriched in cluster 4 rather than across all three clusters.
Please clarify this representation to ensure accuracy.

We agree that our claims of variable co-expression of *Nkx2-1, Foxa2, Meox1, Ripply3, Ascl1* and *Calca* in clusters 4, 6, and 8 are misleading and have removed the corresponding line. Due to the inclusion of new data (pseudotime, trajectory inference), we decided to leave out some of the former Fig. 1q UMAPs (we retain *Nkx2-1*, *Pax8, Foxa2, and Calca* in Fig. 1o) in the revised manuscript and instead include force directed embeddings of the markers in Figure 2e with more precise text in the corresponding results.

4. Inconsistent interpretation of Ripply3 expression (Fig. 1r):

The manuscript states that "Ripply3 predominates early and gradually diminishes." However, Figure 1r suggests that Ripply3 expression peaks at E10.5 before decreasing.
This discrepancy needs to be corrected.

Due to space limitations in revised Fig. 1, UMAP of Ripply3 expression is omitted from this figure but readily visualized across Eday in revised Supplementary fig. 3 and along the new Eday-driven force directed embedding in revised Main fig. 2, which indeed show that Ripply is transiently expressed in the Ubb lineage (Fig. 2f) although Ripply3 is additionally strongly expressed in cluster 7 representing an earlier developmental (pouch endoderm) stage. Text has been revised accordingly (lines 137-148; 168-175).

5. Disappearance of cluster 0 at E12.5:

Figure 1r suggests that Cluster 0 disappears entirely by E12.5.
The authors should confirm whether Cluster 0 is completely absent at this stage and discuss its biological significance.

We agree that Cluster 0 disappears entirely by E12.5. In the revised version, as explained above, we decided to remove Fig. 1r (to leave place for the Venn diagram and orthogonal validation data shuffled from former Fig. 2).

However, we describe the putative role of cluster 0 cells multiple times in the text. Based on gene expression enrichment overlap, we explain that cluster 0 intersects with both lineages (Fig. 1l and associated text; lines 117-127) and therefore likely represents pharyngeal endoderm. Moreover, based on our new pseudotime and trajectory inference analyses, we further corroborate that cluster 0 likely consists of cells that share features of an immature endoderm stage from which both lineages originate (lines 156-163). We think it is not necessary to explain this further other than it becomes apparent from data in revised Fig. 2 that cells in cluster 0 correspond to pharyngeal endoderm and hence are expected to not co-cluster with cells triggered to terminally differentiate at E12.5.

6. Clarification of numerical values (lines 154-157, first ms version):

The meaning of the numbers in the following sentence is unclear:
"Interestingly, upregulation of Ripply3 restricted to clusters 7 (x 36.6), 8 (x 15.1), and 9 (x 7.1) mimics that of Hoxb1 (cluster 7: 33.9; cluster 8: x 18.4; cluster 9: 7.6), which is specifically expressed in the last most inferior pharyngeal pouch."
Do these numbers represent fold changes, normalized expression values, or statistical measures?
This needs to be explicitly clarified.

It is the test statistic (z-score) underlying the computation of a p-value from the signed t-test for each gene for each group. A higher absolute value score corresponds to a lower p-value. As described in the methods, it was obtained from scanpy rank_genes_groups. We now also

briefly clarify the use of z-score in the Results section (line 144-146) and Methods section (lines 723-728) in the text. Notably, there is swap in the values, it should read (corrected in revised paper): “Interestingly, upregulation of Ripply3 restricted to cluster 7 (z-score: 33.9), cluster 8 (15.1) and cluster 9 (7.1) mimics that of Hoxb1 (z-score in cluster 7: 36.6; in cluster 8: 18.4; cluster 9: 7.6), the expression of which is restricted to the most inferior pharyngeal pouch during pharyngeal segmentation”.

7. Interpretation of UMAP data in Supplementary Fig. 2:

Supplementary Figure 2 presents UMAP plots showing gene expression at different time points, but the meaning of the gray dots is unclear.
If these dots represent missing data points, I suggest removing them to make the plots clearer.

The gray dots represent cells present in the dataset from timepoints other than the one indicated in the plot. We have visualized it in this manner, so it becomes evident which cells in the context of the overall shape of the UMAP express genes at each individual time point. We think this is sufficiently explained in the revised Supplementary Fig. 3 legend (note former Suppl Fig 2 is renumbered in the revised manuscript).

8. Misleading conclusion (lines 154-157, first ms version):

The statement "Cluster 1 likely represents a pool of undifferentiated progenitor cells committed to a thyroid fate" is based on Figure 2c.
However, pathway analysis suggests Cluster 3 is more closely related to Cluster 2, while Cluster 1 is more distant.
I recommend using Figure 3a rather than Figure 2c to support this conclusion.

Thanks for this remark which we agree to. We deleted this sentence in favor of (revised lines 133-134): “Most cells in clusters 1-3 co-express *Nkx2-1* and *Pax8* consistent with thyroid progenitor cells that eventually will develop into follicles (Fig. 1c, d)”; and (revised lines 211-212): “…are enriched progressively along with thyroid differentiation i.e. clusters 1→ 2→ 3 (Table 1a; Fig. 3a, b)”.

9. Visualization improvement for figure 3c:

Figure 3c is difficult to interpret.
Consider revising the format to make the data more intuitive.

Agreed, we exchanged this with UMAPs in former Supplementary fig. 5, upgraded to Main Fig 3c and re-plotted for improved visualization.

10. UMAP Visualization of Ki67 Expression in Figure 4a (former Fig. 4a)

The manuscript discusses Ki67 index, but a corresponding UMAP visualization is missing.
Please include a UMAP plot of Ki67 expression.

We have now included a UMAP plot of *Mki67* in revised Supplementary fig. 5, which assembles all cell proliferation-related data in the revised manuscript. Notably, dot plots of Mki67 (originally presented along with Foxa1/2 data) is provided along with the UMAP embedding of Mki67 (Supplementary Fig. 5e, f).

11. Logical flow of the Results section:

The results section lacks clear organization and jumps between different topics.
For example, the text first discusses thyroid clusters, then abruptly shifts to Ubb clusters (Figures 4g and 4k).
Similarly, the immunofluorescence results are not structured systematically.
I suggest reorganizing this section for better logical flow.

We thank the reviewer for this valuable input. Results have now been extensively reorganized to meet this remark, intending to follow a more logical order that better adheres to the natural course of development than in the first manuscript version. Accordingly, the core of former fig. 4 (Foxa1/2) reappears in Main Fig. 7 of the revised manuscript. Switching focus between thyroid and Ubb clusters is nonetheless inevitable since comparing lineage characteristics is an important part of the story throughout.

To summarize, the revised results are now re-organized as follows: Main Figure 1 introduces the scRNAseq data, differential expression of genes and putative cell trajectories; Figure 2 discusses pseudotime analysis and trajectory inference along with complementary *in silico* knockouts and IF; Figure 3 focusses on the thyroid differentiation, inferring gene enrichment, GRNs and RNAscope validation data; Figure 4 presents corresponding findings in the UBB lineage; Figure 5 presents findings corroborating the basement membrane (BM) reorganization in the UBB prior to merging with the thyroid primordium; Figure 6 presents the cell-autonomous breakdown of laminin in the UBB BM followed by mixing of lineage cells; Figure 7 discusses Foxa1/2 and their role in disambiguating C-cells from precursors; Figure 8 discusses EMT of C-cell precursors and the phenotype post thyroid-UBB fusion; and Figure 9 links findings to a subtype of thyroid carcinoma.

12. Contradiction in Foxa1/Foxa2 expression (Fig. 4o):

The manuscript states:
"The large proportion of cells with undetectable or low Foxa1/Foxa2 expression likely reflects permanent repression in thyroid lineage cells and transient downregulation in the fourth pharyngeal pouch."
However, Figure 4o shows that Foxa1/Foxa2 are still expressed in thyroid clusters at E12.5, sometimes even higher than at E9.5.
This inconsistency should be resolved.

The cited statement is still valid, however, we agree with the reviewer that this dot plot visualization was misleading: cluster 1 is dominated by cells at E9.5 and E10.5 (see new Fig.

1i-k). There are very few cells at E12.5 in cluster 1, and, consistent with cluster 1 being a less mature cluster, the few E12.5 cells in cluster 1 are less mature along the thyroid lineage and therefore still express Foxa1/2. Since there are so few cells and the few that exist are dominated by high Foxa1/2 expression at E12.5 in Cluster 1, the mean expression and the fraction of cells expressing Foxa1/2 cells at E12.5 in cluster 1 is high.

Nonetheless, we modified former Fig. 4o to show only the expression of Mki67 (now in Supplementary Fig. 5e), the main reason being transfer of Cell cycle and Ki67 data to Supplementary. Further, we replaced Foxa1/2 dot plots in favor of separate UMAP embeddings across Eday in revised Fig. 7h and g, which convincingly show different expression patterns of Foxa1 and Foxa2 between clusters/lineages and across Eday, accompanied by revised text considering this (lines 400-404).

13. Experimental validation of Foxa2's role in Ubb development:

While bioinformatics analysis suggests Foxa2 regulates Ubb differentiation, this needs experimental validation.
I strongly recommend functional in vivo or in vitro experiments to support this claim.

It is difficult to meet this recommendation because 1) there are currently no suitable transgenic models that allows targeted deletion of Foxa2 specifically in the developing Ubb and 2) there is no robust *in vitro* system that allows investigation of Ubb/C-cell differentiation. As discussed above in the rebuttal to Reviewer #3, embryonic tissue explants might be a way forward to address this question, but this technology is far from being a standard procedure and such optimization would go beyond the scope of this study.

Probably the best option to address this issue in vivo, in view of present findings, we think *Cg-Tg(Prdm1-cre)1Masu/J* (Jackson) might be a plausible Cre driver for targeted deletion of Foxa2 by recombination with *Foxa2*^{tm1Khk}*/J* (Jackson) in Ubb/C-cell precursors. However, taking into account cryorecovery, breeding and basic characterization of homozygous mutants, preliminary results is at least one year ahead and thus out of scope for the current revision.

However, we would like to point out that we have previously investigated the role of Foxa1 and Foxa2 using TT-cells derived from a human medullary thyroid carcinoma (MTC). We showed by RNAi experiments that Foxa1 but not Foxa2 promotes mitogenesis and, moreover, that Foxa2 but not Foxa1 is transiently downregulated in vivo in MTC cells that dedifferentiate and undergo EMT; Foxa2 reappears in metastatic sites characterized by the reverse MET process (DOI: 10.1242/dev.126581). It was also previously shown by in vitro assays that Foxa2 stimulates the calcitonin/calcitonin gene-related peptide gene enhancer in a rat MTC cell line (DOI: 10.1074/jbc.M406659200). This is in line with our finding in the present study, that Foxa2 but not Foxa1 is downregulated in the dedifferentiated MTC tumor component of a mixed (MMFTC) thyroid carcinoma (Fig. 9). We added a short comment on these aspects in the revised manuscript (lines 515-518).

14. Relevance of "Ubb cells of different maturity" analysis:

The bioinformatics analysis in this section appears weakly connected to the study's main focus.
Consider removing or condensing this part.

We condensed this part and deleted former Supplementary Fig. 10 except panels a and b which are transferred to revised Fig. 7 as a means of distinguishing terminally differentiated C-cells from C-cell precursors, a main theme of the final parts of results. Please note that Fig. 7a is truncated (keeping the nine top genes with the highest scores) to fit in the figure; we believe leaving out some upregulated genes does not influence the overall profiling and distinction of Calca+ cells vs Calca- cells.

15. CDH1 and C-cell localization - Validation:

Figure 5 immunofluorescence and Supplementary Figure 13 immunohistochemistry suggest that CDH1-expressing cells may be C-cells.
This should be confirmed using dual-color immunofluorescence or single-cell co-expression analysis of CDH2 and C-cell markers.

Unfortunately, available anti-calcitonin and anti-Cdh2 antibodies do not allow double immunostaining. In the revised manuscript, we show that CDH2+ cells attain a parafollicular or intraepithelial position (revised Fig. 9b), i.e. similarly to C-cells in the adult mouse thyroid, and that CDH2 and CALCITONIN are both expressed in the TT cell line derived from human MTC (revised Fig. 9c). Moreover, we pinpoint a delayed onset of CDH2 expression in the developing Ubb (revised Fig. 8o, p) and refine the localization of CDH1^{low}/CDH2^{high} and CDH1^{high}/CDH2^{low} cells in the thyroid lobe (revised Fig. 8q, r-r´´) consistent with differential expression of *Cdh1* and *Cdh2* in Ubb clusters (revised Fig. 8k). Collectively, these findings arguably indicate Ubb/C-cell lineage cells express Cdh2 both during thyroid organogenesis and in the adult gland and that Cdh2 expression is retained in C-cell-derived tumor cells.

16. Validation of ECM differences between thyroid and Ubb lineages (former Fig. 6):

Figure 6 suggests distinct ECM compositions, but this is based solely on bioinformatics data. Morphological validation (e.g., immunofluorescence for ECM proteins) should be provided.

We revised this part extensively by narrowing down single-cell data presentation in favor of collagen I and collagen IV (revised Fig. 5b and Supplementary Fig. 9a) and, importantly, with validation of in vivo expression pattern of Col1a2 and Col4a2 by RNAscope (revised Fig. 5c-f and Supplementary Fig. 8) and immunofluorescence of differential Col4a2 expression pattern (revised Fig. 5g-m), which confirm and extend bioinformatics data of major differences between thyroid and Ubb cells. This makes a substantial contribution to the revised manuscript.

Notably, immunostaining with an anti-collagen type 1 antibody was inconclusive due to high level of unspecific labeling in nearly all embryonic tissues and therefore considered unreliable

(data not shown). Keeping Col12a1 and Col26a1 in Fig. 5b is intended for comparison of cluster-specific expression.

17. Manuscript Structure and Focus:

The manuscript covers too many topics, ranging from thyroid and C-cell development to thyroid cancer pathology.
Some sections lack experimental validation, and the number of figures is excessive.
I suggest streamlining the manuscript, focusing on a clear central hypothesis, and ensuring all key claims are well-supported by experimental data.
Additionally, some figures appear in a disordered manner, making it difficult to follow the results logically.
The manuscript would benefit from restructuring for clarity and coherence.

As indicated by rephrased subheadings, we have reorganized the entire Results section to improve clarity and coherence. Indeed, the results of validation experiments made it easier for us to follow a more logical order of presentation than in the first version. All main and supplementary figures are restructured and refined to conform with this ambition and the journal´s standard. Thanks to this recommendation and effort, we believe the R1-version of the manuscript is much improved.

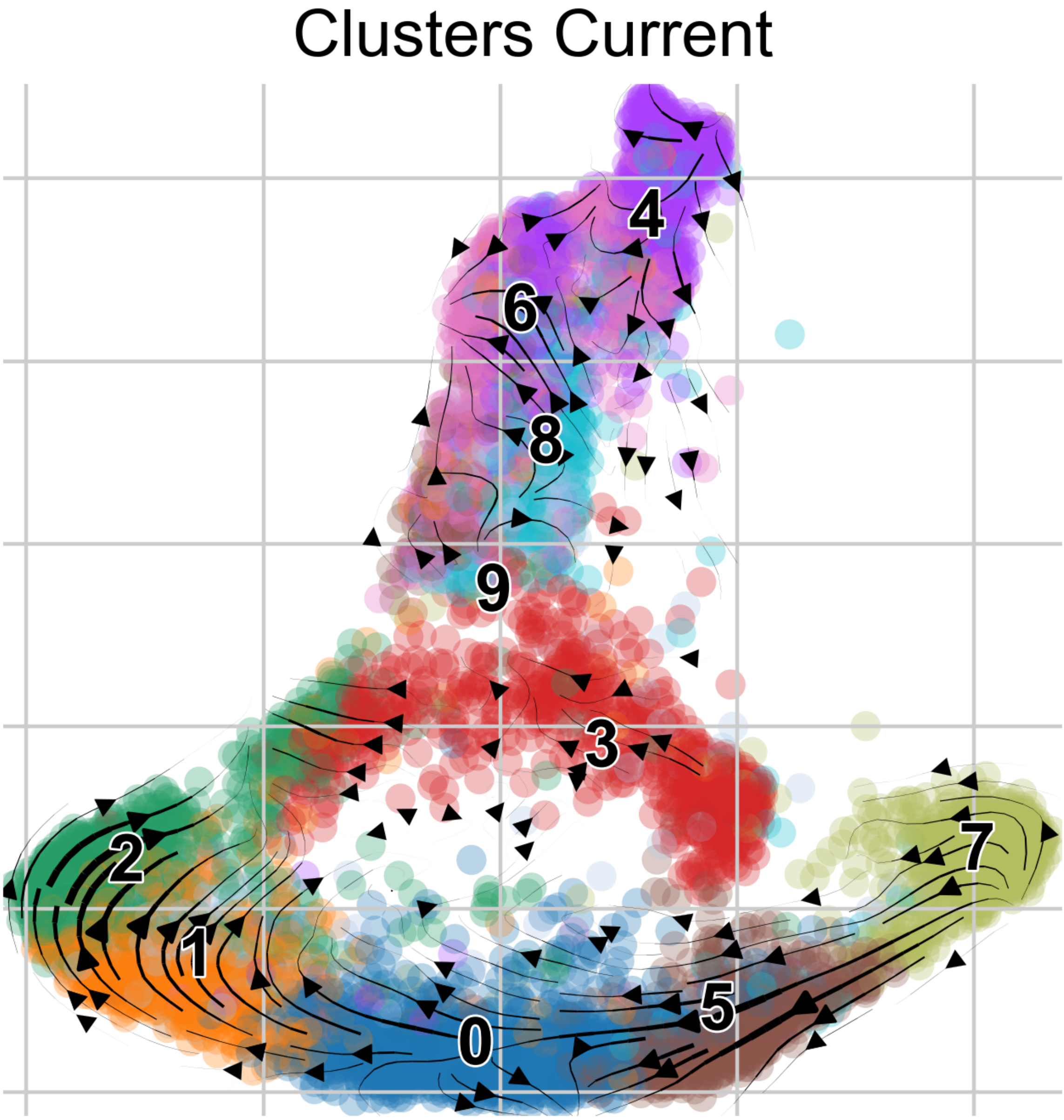

Review Fig. 1.

Cell streamlines inferred with a combined weighted velocity and connectivity kernel with the former inferred from scVelo's dynamic velocity inference model from spliced and unspliced counts and the latter inferred from the gene expression-based cell neighborhood connectivity matrix. Cells are colored by the clusters from Fig. 1. We observe cell streamlines in directions inconsistent with embryonic development

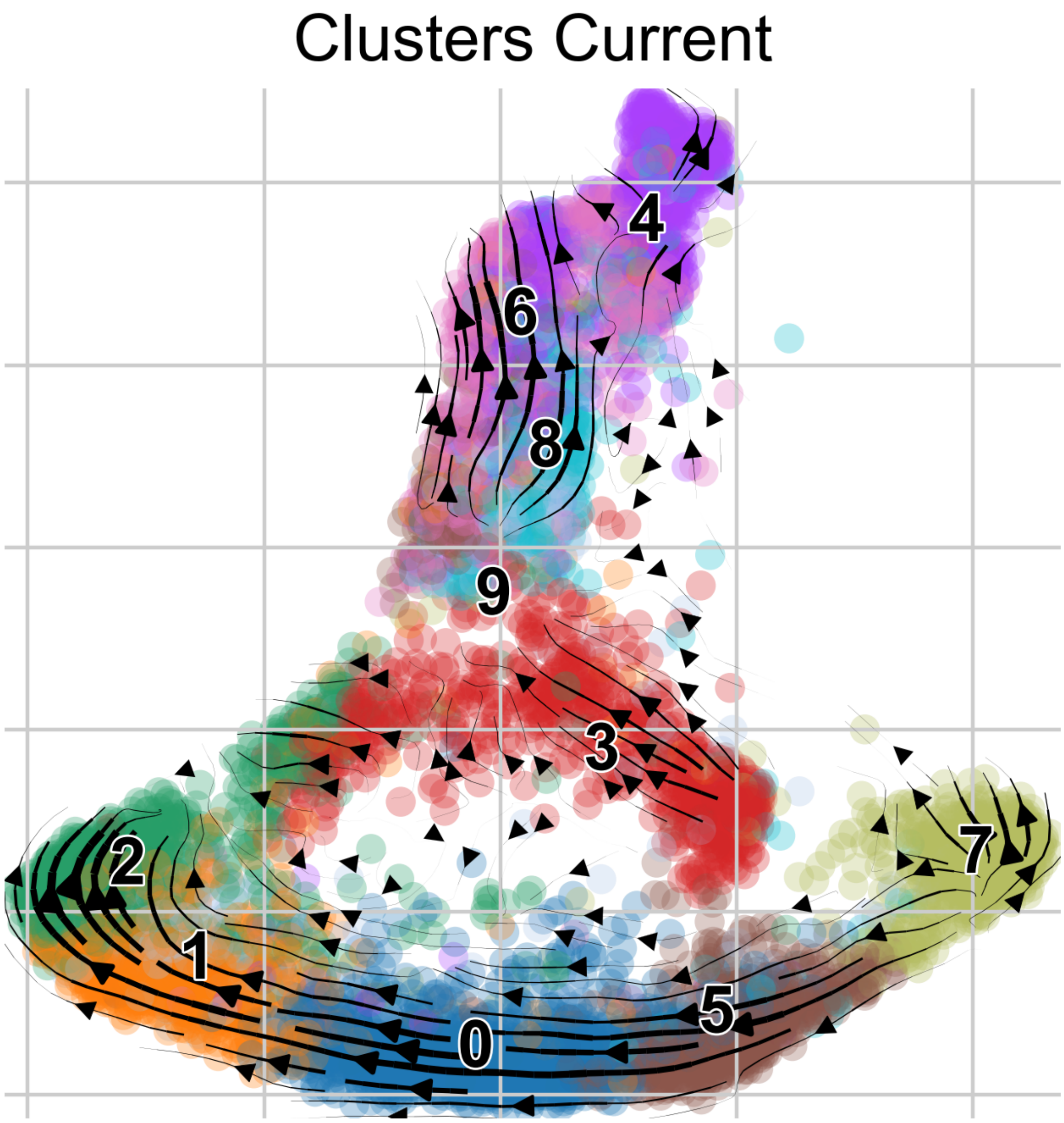

Review Fig. 2

Cell streamlines inferred from Cytotrace using development potential measured by transcriptional diversity to align cells along pseudotime. Cells are colored by the clusters from Fig. 1. We observe cell streamlines in directions inconsistent with embryonic development

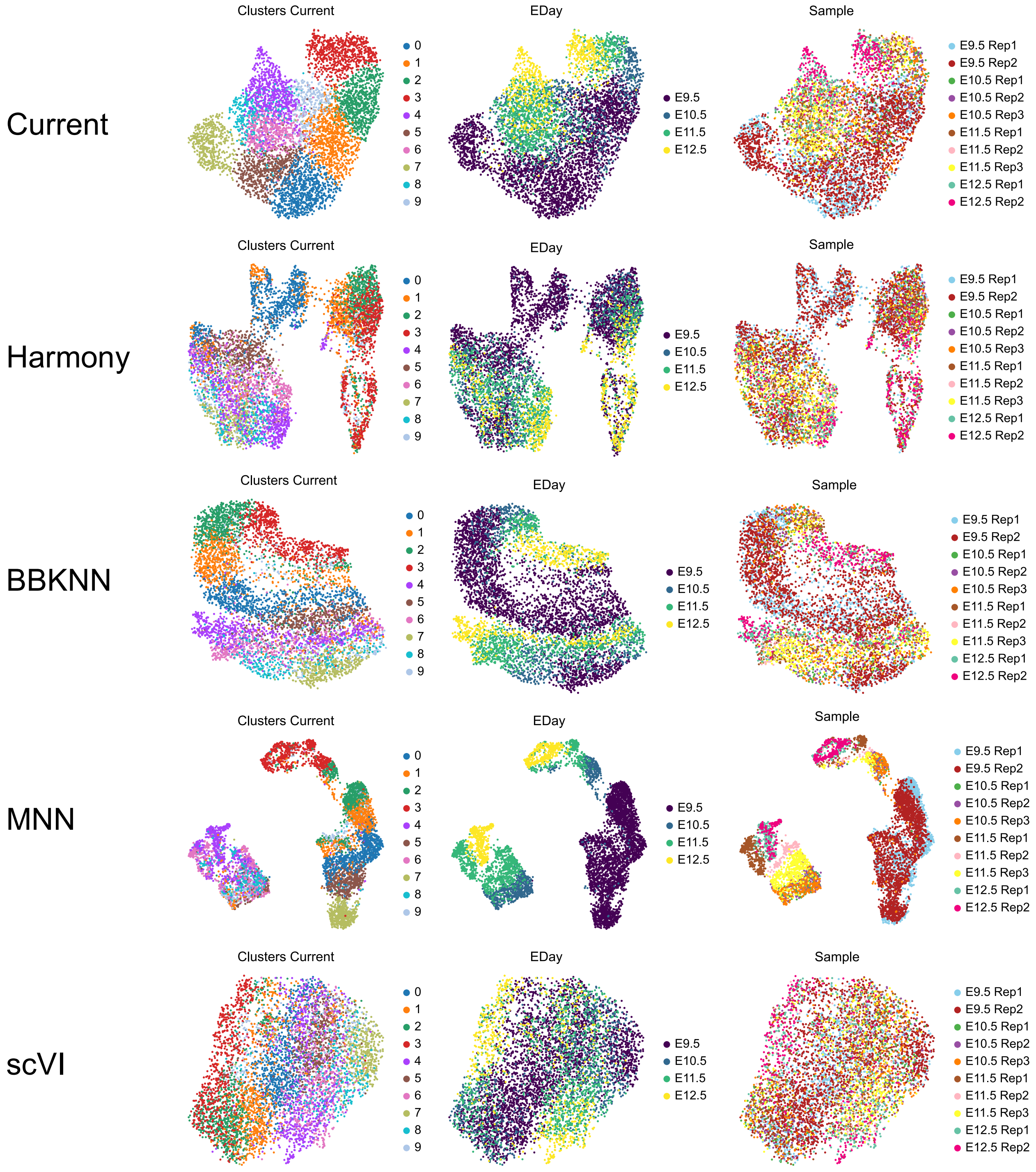

Review Fig. 3

Comparison of UMAP embeddings with batch correction performed using our current approach (combat), Harmony, BBKNN, MNN, and scVI. Cells are colored by clusters (from Fig. 1), Eday and Sample.

Response to Reviewers, NCOMMS-24-66011B, 2nd revision.

We sincerely thank all reviewers for constructive criticism and supportive comments on the original manuscript as well as after the first round of revision.

The present revision deals mainly with Reviewer #3´s remaining points of criticism, as also requested by editor. For this purpose, some sections and paragraphs in Results have been extensively revised with new data being added. This can be found mainly on page 8 (lines 225-238) page 9 (lines 256-278) and page 12 (lines 349-351), also comprising the following new or altered figures/tables/datasets:

- Fig. 3g-i: New (replacing former Fig. 3g-i)
- Fig. 9p: New (former Fig. 9p-q are renumbered and one image is slightly cropped to fit)
- Supplementary Fig. 4a-h: New
- Supplementary Fig. 5a-c: Revised, b with minor additions related to Nkx2-1 in thyroid-GRN, a and c edited by format but not content (to allow revision/increased size of a)
- Supplementary Fig. 6a-e: New
- Supplementary Fig. 7a-g: New
- Supplementary Fig. 11a-d: New (previous/remaining Supplementary Figures are renumbered accordingly)
- Supplementary Dataset 3: New (former ditto renumbered to Dataset 5)
- Supplementary Dataset 4: New
- Supplementary Dataset Legends: Revised
- Supplementary Tables 1-3: New

Since our further validation of the computed model mainly involves animal experiments, we want to highlight that, in total, four mutant mouse strains have now been used in this study:

1. Homozygous *Pax8* null (in original manuscript)
2. Heterozygous *Nkx2.1* null (in original manuscript)
3. Heterozygous *Nkx2.1-CreERT2* (in second revision)
4. Heterozygous *Tg-CreERT2;BrafCA* (in second revision)

The rationale for choice of mutants, performed analyses, and interpretation of results are detailed below specifically addressing Reviewer #3´s issues of concern.

We further verified some obtained scRNAseq data of particular interest with RNAScope/Multiplex double fluorescent monitoring of gene expression in embryonic (Heyl, Pmp22) and adult (Pax8, Tcf4) thyroid tissues.

In support of validation experiments, we also provide additional gene lists (datasets, tables) required as a basis and for the interpretation of new findings related to Nkx2-1 and Heyl.

Since these supplements lengthened the Results section, we have edited the entire manuscript text, primarily intending to improve and clarify but also in the interest of brevity.

As requested by Reviewer #4, the Discussion now also contains a paragraph (page 23, lines 684-695) addressing EMT and migrating phenotypes in relation to recent findings of myeloid cell-derived NF-kB signaling involved in follicle formation.

A few more references have been added according to new points of discussion.

<u>Specific response to Reviewer #3</u>

Your comments on the original manuscript: This interesting study by Lobo et al. effectively uses single cell RNA sequencing approach to distinguish thyroid and C-cell lineages, enabling high-resolution insights into gene expression patterns and lineage-specific trajectories. The manuscript highlights specific transcription factors and regulatory networks, shedding light on the molecular mechanisms guiding thyroid and ultimobranchial body development. This helps understand how gene regulatory cues direct lineage commitment and cellular differentiation. Through gene expression mapping at various embryonic stages, the study identifies lineage-specific changes over time, highlighting the dynamics of cellular differentiation and thyroid organ formation.

While I observe a highly detailed descriptive framework, there is weakness in this study, which is the use of experimentally unvalidated in silico knockout models interpreted as an additional layer of validation for identifying critical genes in the thyroid and Ubb lineage commitment. These models do not perform at the level of trust, and need be either additionally validated with orthogonal experimental approaches (at least some) or discussed with a warning. The performance of CellOracle is very far from 100%. Such predictions from in silico simulations shall be used only to narrow down the hypothesis and run focused experiments, which I expect from the authors during this revision. Overall, the experimental validations of identified regulatory interactions are limited, reducing the direct applicability of single cell transcriptomics-based findings. While the authors provide a lot of correlative gene expression data, numerous conclusions regarding gene function and regulatory network dynamics would benefit from more functional experiments attempted in ex vivo systems, such as explants of developing endodermal derivatives. Also, it would be very interesting to see the phenotypes of and Nkx2-1 mutants at the single cell level, in addition to in silico ko simulations (I really doubt these results will be in coherence).

Your comments on the first revision: I do not believe the authors have sufficiently addressed my comments regarding the need to expand the analysis of mutants and to provide additional functional validations. These points remain important, and I believe the authors could address them in a further round of peer review. While I find the current response largely unsatisfactory, I am not inclined to recommend rejection at this stage.

Thank you for encouraging us to further improve, we appreciate and agree with your sincere concern on the need of functional validations, with the use of suitable mutants.

Recollecting work as presented in the original manuscript, we already verified some in silico findings based on differentially expressed collagens mainly of col4 in thyroid vs Ubb lineage cells, which showed Nkx2-1 dependence of basement membrane degradation (in Nkx2-1 heterozygous null) and that the regulated mechanism likely is cell-autonomous (in homozygous Pax8 null). Since Nkx2-1 null mice either are athyreotic (homozygous) or

thyroid organogenesis is much delayed (heterozygous, this study) we decided to expand this part of the paper aiming to validate predicted Nkx2-1 targets as suggested from the computed thyroid-GRN. To this purpose, we used Nkx2.1-CreERT2+/- mice already available in the lab and which have been instrumental inferring Nkx2-1 gene dosage as critically permissive for BrafV600E-driven thyroid tumorigenesis (Schoultz et al iScience, 2023; DOI: 10.1016/j.isci.2023.107071). In the newly revised manuscript, we show that Pax8 and Tcf4, both being predicted top-ranked thyroid lineage drivers (Supplementary Dataset 1) and directly targeted by Nkx2-1 in the predicted thyroid-GRN (Fig. 3e; Supplementary table 1 NEW), are downregulated in response to monoallelic inactivation of Nkx2-1 in thyroid follicular cells (Supplementary Fig. 4c-h NEW).

Notably, we performed this experiment on adult mice to increase relevance to thyroid physiology. To the best of our knowledge this is the first time Pax8 is proven to be a downstream target, directly or indirectly, of Nkx2-1 in vivo, which thus conforms with the GRN model and suggests a broader repertoire than indicated by the limited number of predicted Nkx2-1 target genes (Supplementary tables 1-3 NEW). Co-expression of Nkx2-1 and Pax8 and regulation of the latter by Nkx2-1 was deduced from double fluorescence in situ hybridization, which also revealed markedly different transcript levels of Nkx2-1 and Pax8 in individual thyrocytes both within and between follicles particularly in wildtype mice and less so in mutants. This observation is consistent with the expression pattern in the computed model, altogether indicating that thyroid cell diversity is pronounced and presumably hard-wired during embryonic development. Notably, synergism with other transcription factors (TFs) is well-established but acting through another TF as Pax8 and Tcf4, potentially by means of transactivation as suggested from the computed model, is new for Nkx2-1 in thyroid cells. All of this is reported and briefly discussed in the revised manuscript.

Secondly, based on its central position in the predicted regulatory network, we narrowed down our interest to Heyl, a downstream mediator of Notch signaling, which showed a broader involvement than most other enriched thyroid TFs in the computed model. Notably, nothing is known before of Heyl in the thyroid. In the current revision work, multiplex staining validated co-expression of Heyl and Pax8 in a subpopulation of thyroid progenitors, i.e. confirming in silico observations, and that additional Heyl+/Pax8-/Nkx2-1- interstitial cells (of yet unknown identity) appear in the embryonic thyroid and adult gland (Fig. 3g-I NEW; Supplementary Fig. 6a-e NEW; Supplementary Dataset 3 NEW). These findings thus support the hypothesis that Heyl is a plausible new player in thyroid development and potentially also in the differentiated thyroid gland. To get further insight, we employed a genetic mouse model of thyroid cancer recently developed by us (Schoultz et al DMM 2022; DOI: 10.1016/j.isci.2023.107071). In this model, thyroid cells expressing mutant Braf become neoplastic and dedifferentiate although Nkx2-1 expression maintains. We now show in the revised manuscript that thyroidal Heyl expression is strongly increased in thyroid tumor tissue and that Heyl turnover changes profoundly in response to a mutant Braf kinase inhibitor (Supplementary Fig. 7a-g). Although these experiments do not reveal a potential mechanistic role of Heyl in thyroid tumorigenesis, they confirm that upregulation of Heyl is a consequence

of oncogenic transformation of thyroid follicle cells and suggest a possibility that Braf mutant cells might recover some features of the embryonic thyroid progenitors.

Lastly, related to characterization of laminin complex constituents in the computed model, we confirmed thyroid-specific mRNA expression of Pmp22 confined to Pax8+ embryonic thyroid cells by double fluorescent staining (Supplementary Fig. 11a-d NEW). The longitudinal expression pattern in vivo of Pmp22 similarly to that of Nkx2-1 and Pax8 is consistent with multiple possible functions along the course of thyroid organogenesis comprising lineage propagation, branching morphogenesis and terminal differentiation. These results thus suggest that Pmp22 is a new thyroid developmental gene. To pursue this further, a Pmp22-floxed mouse strain available at GemPharmatech (C57BL/6JGpt-*Pmp22*^{em1Cflox}/Gpt) suggests conditional knockout is feasible. However, to the best of our knowledge this floxed strain has so far not been employed in any published research reports. Experiments would require extensive work with basic characterization which we think is more suitable for a separate study.

Specific response to Reviewer #4

Your comments on the first revision: The revision addresses my earlier concerns and substantially strengthens the manuscript. The additions include (i) new pseudotime/trajectory analyses (Palantir and moscot implemented within CellRank2), with an explanation of the limited performance of scVelo and CytoTRACE on these data; (ii) added RNAscope, immunofluorescence and Western blot validations at key embryonic stages; and (iii) a clearer, developmentally ordered presentation of Results with an extensive figure/legend re-organization. These changes improve the linkage between single-cell inferences and in vivo morphogenesis.
Minor recommendation for the Discussion.
Please add a brief paragraph situating your EMT-related observations within recent literature on epithelial plasticity in thyroid development, e.g., PMID 38057310. That study reports an NF-κB–activated thyrocyte subpopulation with partial mesenchymal features and shows that myeloid-derived TNF-α promotes thyrocyte migration and folliculogenesis in zebrafish/mouse. A short comparison would help readers: (i) migrating thyrocytes in postnatal/embryonic contexts vs. UBB/C-cell precursors in your embryonic fusion model; (ii) upstream cues (TNF-α–NF-κB axis in thyrocytes) vs. the GRNs and basement-membrane remodeling you define; and (iii) whether the states you observe align best with EMT, collective migration, or a hybrid epithelial/mesenchymal program (your response already discusses collective migration vs. EMT timing). Two to three sentences would suffice; no new experiments are needed.
With the above brief addition to the Discussion and routine checks, I support acceptance.

Thank you for appreciating our efforts to improve the manuscript and highlighting the NCOMMS paper from 2023 by Yang et al on the influence of myeloid-derived cytokines on

postnatal thyroid folliculogenesis. As indicated above, we have added a paragraph in the Discussion following your suggestions to clarify some points of concern regarding epithelial plasticity in the present model and put into perspective, hoping you find it sufficient.
